# *WRKY* Transcription Factors Associated With *NPR1*-Mediated Acquired Resistance in Barley Are Potential Resources to Improve Wheat Resistance to *Puccinia triticina*

**DOI:** 10.3389/fpls.2018.01486

**Published:** 2018-10-17

**Authors:** Jing Gao, Weishuai Bi, Huanpeng Li, Jiaojiao Wu, Xiumei Yu, Daqun Liu, Xiaodong Wang

**Affiliations:** ^1^College of Plant Protection, Biological Control Center for Plant Diseases and Plant Pests of Hebei, Hebei Agricultural University, Baoding, China; ^2^College of Life Sciences, Hebei Agricultural University, Baoding, China; ^3^Graduate School of Chinese Academy of Agricultural Sciences, Beijing, China

**Keywords:** *WRKY* transcription factors, *NPR1*, acquired resistance, barley, wheat, *Puccinia triticina*

## Abstract

Systemic acquired resistance (SAR) in *Arabidopsis* is established beyond the initial pathogenic infection or is directly induced by treatment with salicylic acid or its functional analogs (SA/INA/BTH). NPR1 protein and WRKY transcription factors are considered the master regulators of SAR. Our previous study showed that *NPR1* homologs in wheat (*Triticum aestivum* L.) and barley (*Hordeum vulgare* L.) regulated the expression of genes encoding pathogenesis-related (PR) proteins during acquired resistance (AR) triggered by *Pseudomonas syringae* pv. *tomato* DC3000. In the present examination, AR induced by *P. syringae* DC3000 was also found to effectively improve wheat resistance to *Puccinia triticina* (*Pt*). However, with more complex genomes, genes associated with this SAR-like response in wheat and barley are largely unknown and no specific *WRKYs* has been reported to be involved in this biological process. In our subsequent analysis, barley transgenic line overexpressing wheat *wNPR1* (wNPR1-OE) showed enhanced resistance to *Magnaporthe oryzae* isolate Guy11, whereas AR to Guy11 was suppressed in a barley transgenic line with knocked-down barley *HvNPR1* (HvNPR1-Kd). We performed RNA-seq to reveal the genes that were differentially expressed among these transgenic lines and the wild-type barley plants during the AR. Several *PR* and BTH-induced (*BCI*) genes were designated as downstream genes of *NPR1*. The expression of few *WRKYs* was significantly associated with *NPR1* expression during the AR events. The transient expression of three *WRKY* genes, including *HvWRKY6*, *HvWRKY40*, and *HvWRKY70*, in wheat leaves by *Agrobacterium*-mediated infiltration enhanced the resistance to *Pt*. In conclusion, a profile of genes associated with *NPR1*-mediated AR in barley was drafted and *WRKYs* discovered in the current study showed a substantial potential for improving wheat resistance to *Pt*.

## Introduction

Systemic acquired resistance (SAR) is an inducible form of plant defense that confers broad-spectrum immunity to secondary infections beyond the initial infection site. In *Arabidopsis*, SAR is associated with accumulation of the plant hormone salicylic acid (SA) and transcriptional activation of pathogenesis-related (*PR*) genes (Zheng and Dong, 2013). The *Arabidopsis* NPR1 protein (Non-expresser of *PR* genes 1, also known as NIM1 and SAI1) is a master regulator required for SAR. Upon pathogen infection or treatment with SA or its functional analogs 2,6-dichloroisonicotinic acid (INA) and benzothiadiazole (BTH), NPR1 translocates from the cytoplasm into the nucleus, where it interacts with the TGA2 transcription factor to promote expression of multiple *PR* genes (Cao et al., 1994; Delaney et al., 1995; Ryals et al., 1997; Shah et al., 1997; Mou et al., 2003). Overexpression of *Arabidopsis* *NPR1* (*AtNPR1*) in other plant species (e.g., rice and wheat) enhances their resistance against multiple pathogens (Chern et al., 2001; Makandar et al., 2006; Quilis et al., 2008; Gao et al., 2013; Xu et al., 2017). In *Arabidopsis* and rice, several *WRKY* transcription factors (TFs) have been suggested to play important roles in the *NPR1*-mediated SAR. A previous genomic approach has identified several *WRKYs*, including *AtWRKY18*, *AtWRKY58*, and *AtWRKY70*, as regulatory nodes in the transcriptional network of SAR in *Arabidopsis* (Wang et al., 2006). On the other hand, *OsWRKY3* and *OsWRKY71* in rice were reported as upstream genes of the rice *NPR1* homolog (*NH1*) (Liu et al., 2005, 2007). Another *WRKY* transcription factor in rice, *OsWRKY45*, was established as an independent regulator in the SA/BTH signaling pathway (Shimono et al., 2007; Nakayama et al., 2013).

Our previous analysis showed that the wheat NPR1 homolog (wNPR1) interacts with four members of the basic-region leucine zipper (bZIP) transcription factor family (also known as TGA) (Cantu et al., 2013). The interactions between NPR1 and TGAs are critical for NPR1 functioning in *Arabidopsis* and rice (Chern et al., 2001; Després et al., 2003). However, SAR in wheat and barley somewhat differs from that described in model plants of *Arabidopsis* and rice (Wang et al., 2018). In early investigations, SA/INA/BTH treatment of wheat and barley induced a SAR-like response, BTH-induced resistance (BIR), to various pathogens, including *Blumeria graminis* and *Puccinia triticina* (*Pt*) (Görlach et al., 1996; Beßer et al., 2000; Hafez et al., 2014). Other studies established that most of the *PR* genes were not induced by such treatments (Vallélianbindschedler et al., 1998; Molina et al., 1999). Moreover, another group of BTH-inducible genes, such as the wheat chemical-induced (*WCI*) and barley chemical-induced (*BCI*) genes, may be responsible for the enhanced resistance to various pathogens during BIR.

In another study, *Pseudomonas syringae* pv. *japonica* (*Psj*) or *Xanthomonas translucens* pv. *cerealis* (*Xtc*) induced systemic immunity (SI) against secondary infection of *Xtc* in uninfected barley leaves (Dey et al., 2014). The findings of the same study also indicated that SI was not associated with barley *HvNPR1*, or local or systemic accumulation of SA, but with several *WRKY* and *ERF* transcription factors.

As a third form of SAR-like response in barley, acquired resistance (AR) to the secondary pathogen *Magnaporthe oryzae* (*Mo*) was induced in the area adjacent to the initial infection of *P. syringae* pv. *tomato* DC3000 in a *PR* genes-induced manner, but such resistance was not systemic (Colebrook et al., 2012). The levels of both free and conjugated SA were significantly upregulated in barley leaves infiltrated with *P. syringae* pv. *syringae* (Vallélianbindschedler et al., 1998). In our previous research, the induction of several barley *PR* genes, including *HvPR1b*, *HvPR2*, *HvPR3_Chit2a*, and *HvPR5_TLP6*, was significantly associated with the expression level of *NPR1* in transgenic lines overexpressing wheat *NPR1* (wNPR1-OE) or suppressing barley *NPR1* (HvNPR1-Kd) during the *P. syringae* DC3000-triggered AR (Wang et al., 2016). However, genes associated with these biological processes are largely unknown and no specific *WRKYs* has been reported to be involved in this SAR-like response in barley.

In the present study, we established that AR induced by *P. syringae* DC3000 also improved wheat resistance to *P. triticina* (*Pt*). In addition, the AR responses of the barley transgenic lines wNPR1-OE and HvNPR1-Kd to the *Mo* isolate Guy11 were evaluated. Further, transcriptome analysis of these transgenic lines during AR response was carried out. The downstream genes of *NPR1* were identified based on their expression profiles. The inductions of several transcription factors displayed significant association with the expression of *NPR1* during AR. Three differentially expressed *WRKY* genes, identified in our transcriptome database, showed a considerable potential for improving wheat resistance to *Pt*.

## Materials and Methods

### Plants and Pathogens

A transgenic line of barley overexpressing wheat *wNPR1* (wNPR1-OE) and a transgenic line with suppressed barley *HvNPR1* (HvNPR1-Kd) under maize Ubiquitin promoter in the background of cultivar “Golden Promise” were derived from previous studies (Dey et al., 2014; Wang et al., 2016). The wild-type plants severed as control. The fully expanded third leaves from the experimental plants were used for *P. syringae* pv. *tomato* DC3000 infiltration and subsequent *M. oryzae* (*Mo*) inoculation. Briefly, *P. syringae* DC3000 was grown in KB medium with Rif antibiotics and was then diluted to OD600 = 0.5 in sterile water. Third leaves were inoculated with a 1-mL needless syringe by pressure infiltration of bacterial suspensions through the leaf abaxial surface. The borders of the infiltrated region were marked using a marker pen. Control seedlings were infiltrated in the same way with sterile water. After bacterial inoculation, seedlings were maintained at a constant temperature of 23°C to facilitate bacterial growth. Samples for RNA extraction were collected from regions adjacent to the infiltration from the transgenic lines and the wild-type plants 48 h post-inoculation (hpi) after a clear cell death phenotype triggered by *P. syringae* DC3000 infection was observed (Colebrook et al., 2012; Wang et al., 2016).

The same adjacent regions were also used for *Mo* inoculation to evaluate the degree of acquired resistance in the transgenic lines and the wild-type plants. *Mo* isolate Guy11 was grown on complete medium for 11 days at 25°C under a 16/8-h light-dark cycle. Ten microliters of the conidia suspension (5.0 × 10^5^ spores per milliliter) containing 0.05% Tween-20 was dropped to the press-injured spots on the adjacent region and then wrapped with cellophane tape. The plants were kept in a mist chamber at 25°C in the dark for the first 24 hpi and then transferred to a growth chamber at 23°C and 80% humidity under a 16/8-h (light/dark) photoperiod. The inoculated leaves were photographed 5 days later. The size of the lesion caused by *Mo* on each leaf was measured. The assay for each treatment and phenotype combination consisted of at least six biological replicates. Data were transformed to restore normality and Dunnett’s test was performed using SAS software v9.4 (SAS Institute, Cary, NC, United States).

A similar approach was employed to induce AR in wheat using *P. syringae* DC3000 infiltration. Water infiltration served as negative control. Fully expanded secondary leaves from seedlings of the wheat susceptible line “Thatcher” were used in the experiments. Urediniospores of highly virulent *Pt* pathotype THTT were sprayed on the region adjacent to the *P. syringae* DC3000 infection area 2 days post-infiltration. Inoculated wheat plants were maintained in a moist chamber at 18°C for 16 h in the dark and were next transferred into a growth chamber with 16 h light at 23°C and 8 h darkness at 18°C. The phenotype of leaf rust was recorded at 10 dpi. The percentage of *Pt* sporulation area in the corresponding region for each leaf was determined using ASSESS (version 2.0) image analysis software for plant disease quantification from the American Phytopathology Society (Lamari, 2008; Zhang et al., 2017). The whole experiment was repeated twice and each repeat consisted of 11–14 biological replicates. The data were transformed to restore normality and general linearized model (GLM) ANOVA was conducted using SAS software version 9.4 (SAS Institute, Cary, NC, United States).

### RNA Isolation and qRT-PCR

RNA samples for RNA-seq and qRT-PCR assays were isolated using a plant total RNA extraction kit (Qiagen, Hilden, Germany) following the manufacturer’s instructions. The first-strand cDNA was synthesized using a reverse transcription kit (Clontech, Mountain View, CA, United States). Then, gene expression was quantified as described before (Wang et al., 2016), using the barley elongation factor 1-a (*HvEF1a*, GenBank accession number Z50789) and actin (*HvActin*, GenBank accession number AK362208) as internal references. The qRT-PCR primers, designed for the selected *PR* genes (*HvPR1b*, *HvPR2*, and *HvPR3_Chit2a*, derived from our previous study), *BCI* genes (*HvBCI1*, *HvBCI3*, and *HvBCI7*), and *NPR1* gene, are listed in **Supplementary Table S1**. Further, the amplification efficiency for each pair of primers was calculated using five fourfold cDNA dilutions (1:1, 1:4, 1:16, 1:64, and 1:256). To ensure amplification specificity, dissociation curves for the temperature range from 60 to 94°C were generated for each reaction. The threshold values (Ct) generated from the Roche LightCycler 96 were used to quantify the relative gene expression using the Delta-Ct method as described earlier (Wang et al., 2016). Two independent transgenic lines for each of the wNPR1-OE and HvNPR1-Kd were utilized. Each experiment consisting of 4–11 biological replicates was considered as a block. Calculations of the mean and standard error were performed using Microsoft Excel (Microsoft, Redmond, WA, United States). The data were transformed to restore normality and GLM ANOVA was conducted using SAS software version 9.4 (SAS Institute, Cary, NC, United States).

### RNA-seq

Library preparation and RNA-seq were performed as described in the Illumina TruSeq RNA Sample Preparation Version 2 Guide, the Illumina HiSeq 1000 System User Guide (Illumina), and the KAPA Library Quantification Kit-Illumina/ABI Prism User Guide (Kapa Biosystems) by Novogene Co., Ltd. The sequencing run was performed on a HiSeq 1000 instrument. Then, the sequence reads were mapped on the Ensembl Genomes *Hordeum vulgare* genome sequence (International Barley Genome Sequencing Consortium, 2012) using TopHat 2.0.8 (Trapnell et al., 2009) with default parameter settings and an expected mean insert size of 150 bp. The assembled contigs that were not aligned with the reference genome were annotated as “Novel” transcripts. Further, HTSeq was used to assemble the mapped RNA-seq reads into transcripts to quantify their relative abundance (Trapnell et al., 2010). Differentially expressed genes were identified using the default settings of DESeq2 (Love et al., 2014) and filtered for an FDR-adjusted *P* < 0.05. Statistically significant over-representation of GO categories within differentially expressed genes was determined using the GOseq package (Young et al., 2010). Statistically significant GO terms tested by conditional hypergeometric tests (*P* < 0.05) were considered enriched. Next, heatmaps were generated by MeV software using FPKM values from the RNA-seq database. Hierarchical clustering analysis was performed by the MeV software, based on which the genes with similar expression patterns were clustered. A summary of GO annotation categories was generated using the GOLevel2 Counter in the TBtools software.

### Transient Expression Assay

RNA isolated from barley leaves was utilized for cDNA synthesis using the reverse transcription kit (Clontech, Mountain View, CA, United States). Subsequently, the cDNA sequences of 10 selected *WRKY* genes were PCR-amplified using the primers listed in **Supplementary Table S1**. Initially, the PCR products were cloned into a pGEM-T easy vector (Promega, Madison, WI, United States) and then into a wheat transgenic vector pLGY02, which contained the maize (*Zea mays*) ubiquitin 1 promoter and T-DNA insertion site. The recombinant constructs were transformed into the *Agrobacterium* strain AGL1, and the wheat leaves were subjected to transient gene expression assays as previously described (Lu et al., 2016). Fresh *Agrobacterium* was grown overnight in yeast extract broth (YEB) medium supplemented with Rifampicin and Kanamycin. The bacterial pellets obtained after centrifugation were resuspended in an infiltration buffer containing 10 mM MES, 10 mM MgCl_2_, and 400 μM acetosyringone to an optical density of OD600 = 2.0. For wheat infiltration, the fully expanded secondary leaf of wheat seedlings was infiltrated using a 1-mL syringe without a needle. The border of the infiltration area was marked with a mark pen. Urediniospores of highly virulent *Pt* pathotype THTT were spray-inoculated 4 days post-infiltration. The inoculated leaves were photographed at 10 days post-inoculation. The percentage of *Pt* sporulation area in the infiltration region for each leaf was calculated using ASSESS software v2.0 (American Phytopathology Society) (Lamari, 2008; Zhang et al., 2017). The assay for each gene was repeated at least twice, and each repeat consisted of 5–18 biological replicates. Data were transformed to restore normality and Dunnett’s test was carried out using SAS software v9.4 (SAS Institute, Cary, NC, United States). Raw data for all these conducted experiments were archived in **Supplementary File S1**.

## Results

### AR Triggered by *P. syringae* DC3000 in Wheat Reduces the Severity of *Pt*

**FIGURE 1:**
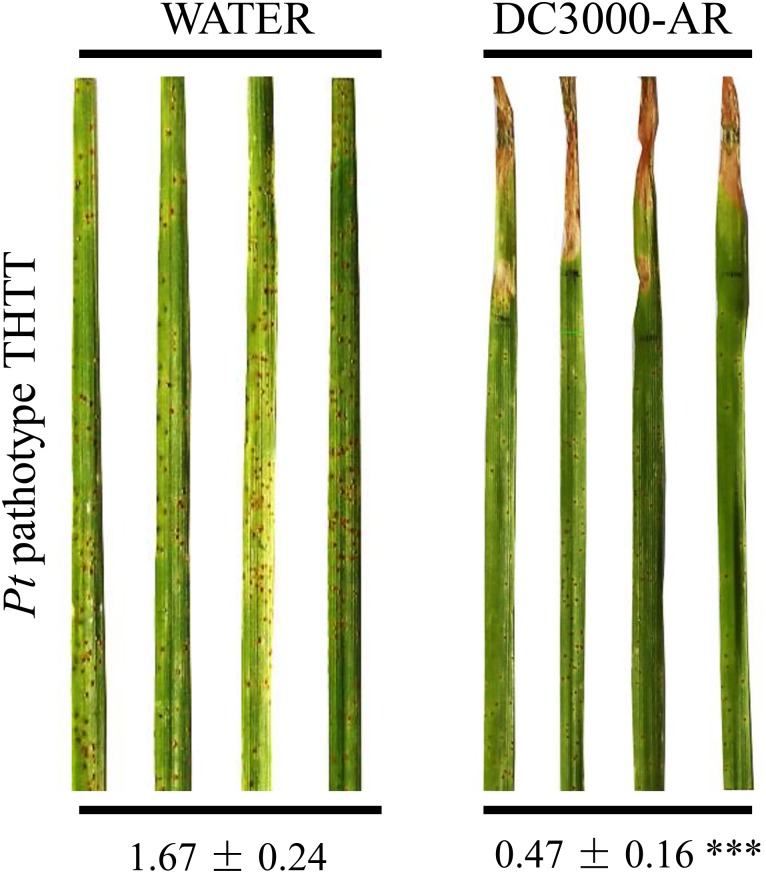
AR triggered by *P*. *syringae* DC3000 in wheat reduces the severity of *Pt.* A highly virulent *Pt* pathotype THTT was inoculated in the region adjacent to either *P. syringae* DC3000 or water infiltration area in wheat leaves of the susceptible line “Thatcher.” The susceptible phenotypes of leaf rust were observed in the region adjacent to both *P. syringae* DC3000 and water mock infiltration area at 10 dpi. The numbers below the images of the leaves represent the average percentages of the *Pt* sporulation areas in the corresponding leaf regions (*n* = 25). Compared with the mock control, a significantly (^∗∗∗^*P* < 0.0001) more enhanced resistance to *Pt* was observed in the region adjacent to the *P*. *syringae* DC3000 infection area. The whole experiment was repeated twice, and each repeat, consisting of 11–14 biological replicates, was considered as a block. The data obtained were transformed to restore normality, and general linearized model (GLM) ANOVA was conducted using SAS software version 9.4.

AR triggered by *P. syringae* DC3000 in barley was considered as a SAR-like response providing broad-spectrum protection against subsequent pathogen challenge (Colebrook et al., 2012). To elucidate whether such AR can be utilized to improve the resistance of other *Triticeae* crops, we examined its effect in the defense reaction of wheat to leaf rust, which is a severe fungal disease in wheat. A highly virulent *Pt* pathotype THTT was used to inoculate the region adjacent to *P. syringae* DC3000 or water infiltration area in the wheat leaves of the susceptible line “Thatcher.” Cell death triggered by *P. syringae* DC3000 was observed 2 days post-infiltration. The susceptible phenotypes of leaf rust were identified in both *P. syringae* DC3000 and water mock treatments 10 days post-inoculation (**Figure 1**). The percentage of *Pt* sporulation area for each leaf was calculated using ASSESS software. Significantly more (*P* < 0.0001) promoted resistance to *Pt* was observed in the region adjacent to the *P. syringae* DC3000 infection area than in the mock control (**Figure 1**).

### Barley AR to *Mo* Isolate Guy11 Is Mediated by *NPR1*

**FIGURE 2:**
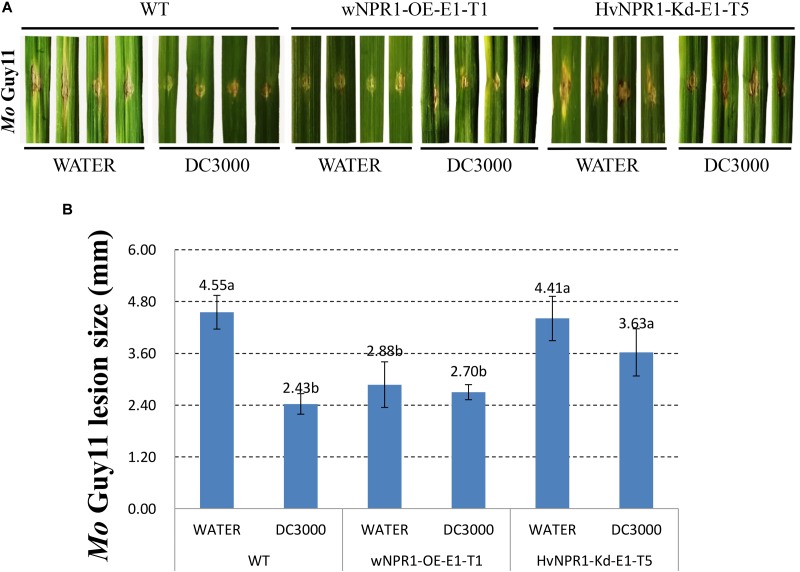
Barley AR to *Mo* isolate Guy11 is mediated by *NPR1.* **(A)** Symptoms of *M. oryzae* isolate Guy11 after inoculation in the region adjacent to *P*. *syringae* DC3000 infiltration area in the wNPR1-OE and HvNPR1-Kd barley transgenic lines and the wild-type plants. Distilled water was mock-infiltrated to serve as a control. **(B)** Quantification of *M. oryzae* lesion size. The assay for each treatment and phenotype combination consisted of at least six biological replicates. The data were transformed to restore normality, and Dunnett’s test (*P* < 0.05) was conducted using SAS software v9.4.

The *Mo* isolate Guy11 was used to determine the extent of AR triggered by *P. syringae* DC3000 in a barley transgenic line overexpressing wheat *wNPR1* (wNPR1-OE), a transgenic line with knocked-down barley *HvNPR1* (HvNPR1-Kd), and wild-type plants (**Figure 2**). The third leaf of barley plants was infected by infiltration with *P. syringae* DC3000 or treated with sterile water as a mock control. *Mo* isolate Guy11 was inoculated in the region adjacent to *P. syringae* DC3000 infiltration area when a cell death was observed 2 days post-infiltration (dpi). Strong AR against *Mo* isolate Guy11 was established in the region adjacent to *P. syringae* DC3000 infection area in the wild-type plants (**Figure 2A**). The size of the lesions caused by the *Mo* isolate Guy11 in the region adjacent to *P. syringae* DC3000 infection area was significantly (*P* < 0.05) lower than that in the mock control (**Figure 2B**). The wNPR1-OE transgenic line had more pronounced resistance to *Mo* infection than the wild-type plants, even in the mock control (**Figure 2**). The AR triggered by *P. syringae* DC3000 in the HvNPR1-Kd transgenic line, was suppressed but not fully eliminated (**Figure 2**), possibly because *NPR1* was not completely removed from the HvNPR1-Kd line. These results indicate that the AR to the *Mo* isolate Guy11, triggered by *P. syringae* DC3000, is mediated by *NPR1*.

### RNA-Seq Analysis Was Applied on wNPR1-OE and HvNPR1-Kd Barley Transgenic Lines During AR Triggered by *P. syringae* DC3000

To explore the gene regulation network during the *NPR1*-mediated AR in barley, we performed RNA-seq analysis on samples collected from the region adjacent to either *P. syringae* DC3000 or water infiltration area in the wNPR1-OE and HvNPR1-Kd barley transgenic lines and the wild-type plants. A number of 4–7 biological replicates for each treatment and genotype combination were sent for 6-Gb RNA sequencing (**Supplementary Table S2**). At least 46 million reads were sequenced (150-bp pair-end) from each sample and mapped on the Ensembl Genomes *H. vulgare* genome sequence with a total of 31,794 genes, including 7,583 “Novel” transcripts. A clear correlation between the gene expression levels of the biological replicates was observed (*R*^2^ > 0.92, **Supplementary Figure S1**). The abundance of accumulated transcripts was estimated using the fragments per kilobase of transcript per million mapped reads (FPKM) value. All raw data were uploaded to NCBI in BioProject PRJNA431836.

### Expression Profiles of *PR* and *BCI* Genes in the *NPR1*-Mediated AR in Barley

**FIGURE 3:**
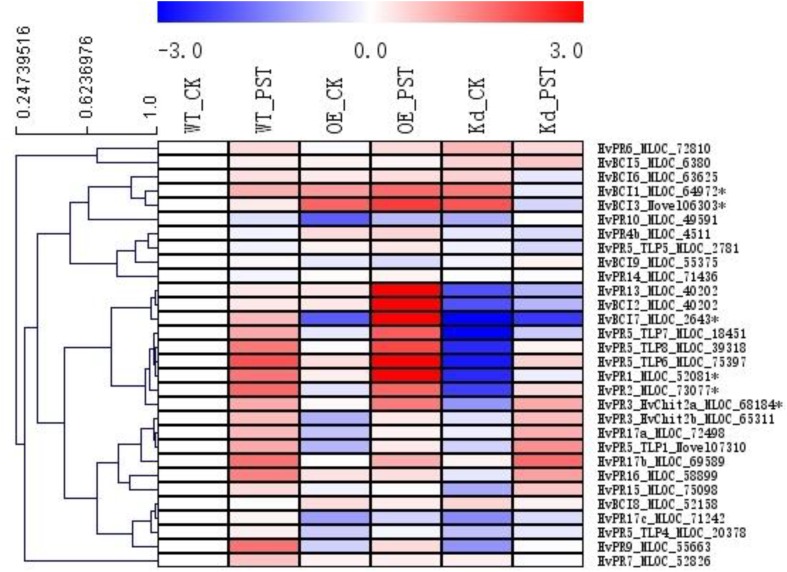
Expression profiles of *PR* and *BCI* genes during the *NPR1*-mediated AR. A heatmap was generated by MeV software using the FPKM values of *PR* and *BCI* genes from the RNA-seq database. Genes with similar expression patterns were clustered using the “Hierarchical Clustering” function of the MeV software. The expression levels of six genes (^∗^asterisk-labeled) were further validated by qRT-PCR assay.

**FIGURE 4:**
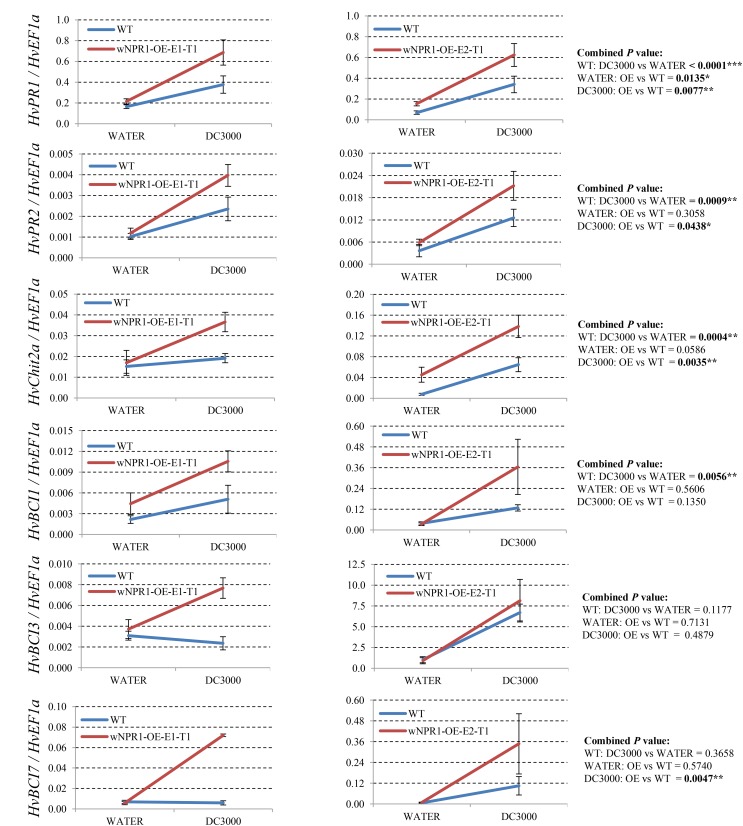
Transcript levels of selected *PR* and *BCI* genes in the wNPR1-OE transgenic lines. Third leaves of wNPR1-OE barley transgenic lines and wild-type plants were infiltrated with water (control) or *P. syringae* pv. *tomato* DC3000. Samples for qRT-PCR assays were collected from the leaf region adjacent to the infection 48 h after inoculation, after a cell death phenotype observed. Using the 2^−ΔCT^ method, the transcript levels were expressed relative to those of the endogenous control *HvEF1a*. Two independent transgenic lines for the wNPR1-OE were used. Each experiment, consisting of 4–11 biological replicates, was considered as a block. Calculations of the mean and standard error were performed using Microsoft Excel software. Data were transformed to restore normality and general linearized model (GLM) ANOVA (^∗^*P* < 0.05, ^∗∗^*P* < 0.01, ^∗∗∗^*P* < 0.0001) was conducted using SAS software version 9.4.

**FIGURE 5:**
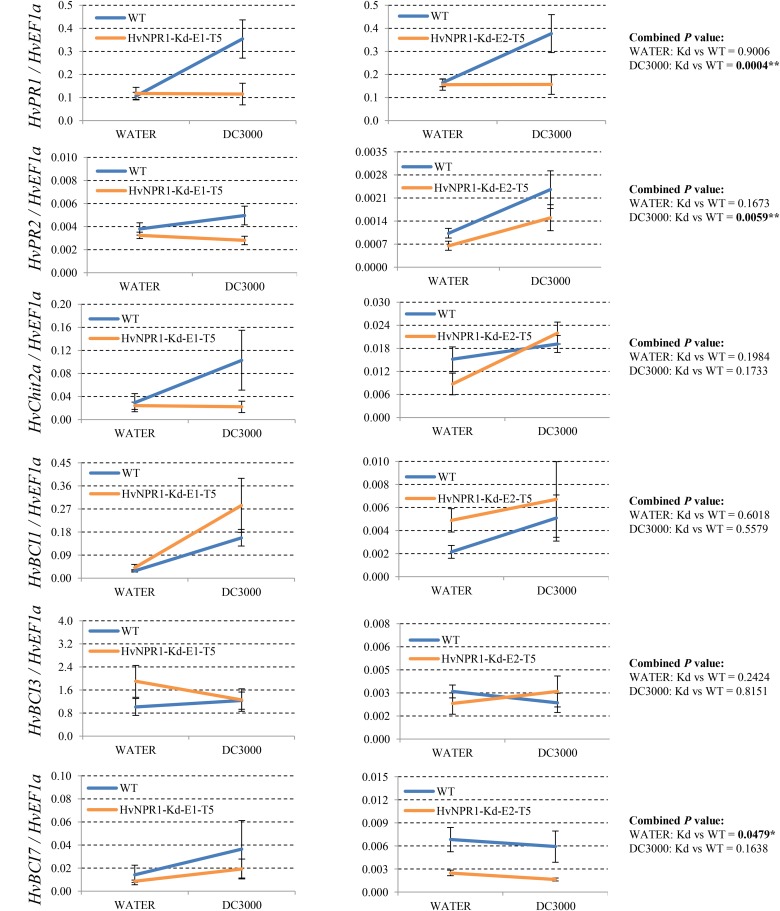
Transcript levels of selected *PR* and *BCI* genes in the HvNPR1-Kd transgenic lines. Third leaves of HvNPR1-Kd barley transgenic lines and wild-type plants were infiltrated with water (control) or *P. syringae* pv. *tomato* DC3000. Then, samples for qRT-PCR assays were collected from the leaf region adjacent to the infection 48 h after inoculation, after a cell death phenotype observed. The transcript levels are expressed relative to those of the endogenous control *HvEF1a* using the 2^−ΔCT^ method. Two independent transgenic lines for the HvNPR1-Kd were used, and, each experiment, consisting of 4–11 biological replicates, was considered a block. Calculations of the mean and standard error were performed using Microsoft Excel software. Data were transformed to restore normality and general linearized model (GLM) ANOVA (^∗^*P* < 0.05, ^∗∗^*P* < 0.01) was conducted using SAS software version 9.4.

Since many of the *PR* genes were reported as downstream genes of *NPR1* during the *P. syringae* DC3000-triggered AR in our previous study (Wang et al., 2016), in the present investigation, we initially checked the expression levels of all the *PR* gene families in our RNA-seq database (**Figure 3**). We noticed that the transcript levels of *HvPR1*, *HvPR2*, *HvPR3_Chit2a*, *HvPR5* (*TLP6*, *TLP7*, and *TLP8*), *HvPR9*, and *HvPR13*, were significantly associated with the expression of *NPR1* as either significantly (*P* < 0.05) higher induced in the wNPR1-OE transgenic line or lower induced in the HvNPR1-Kd transgenic line (**Figure 3**). In contrast, the expression levels of *PR5* (*TLP1*), *PR15*, *PR16*, *PR17a*, and *PR17b* showed induction by *P. syringae* DC3000 but in a *NPR1*-independent manner. Since a group of *BCI* genes were previously reported to be responsible for the BTH-induced resistance in barley (Beßer et al., 2000), we examined the expression patterns of all barley *BCI* genes in our database and found that *HvBCI2* (the same gene as *HvPR13*) and *HvBCI7* were regulated by *NPR1* during the *P*. *syringae* DC3000-triggered AR (**Figure 3**). qRT-PCR assay was carried out to validate the gene expression data obtained from the RNA-seq database. Two independent transgenic lines for each of the wNPR1-OE and HvNPR1-Kd were used and the barley elongation factor *HvEF1a* (GenBank accession number Z50789) were employed as a reference gene. We found that the expression level of *NPR1* in the wNPR1-OE and HvNPR1-Kd transgenic lines was significantly (*P* < 0.01) higher and lower, respectively, than that in the wild-type plants (**Supplementary Figure S2**). In addition, the expression levels of six selected genes, including *HvPR1*, *HvPR2*, *HvPR3_Chit2a*, *HvBCI1*, *HvBCI3*, and *HvBCI7*, were measured by qRT-PCR. In the wild-type plants, significant (*P* < 0.01) inductions of *HvPR1*, *HvPR2*, *HvPR3_Chit2a*, and *HvBCI1*, were observed upon the *P*. *syringae* DC3000 treatment (**Figure 4**). Furthermore, the expression levels of *HvPR1*, *HvPR2*, *HvPR3_Chit2a*, and *HvBCI7*, were significantly (*P* < 0.05) upregulated in the wNPR1-OE transgenic lines by the *P*. *syringae* DC3000 treatment (**Figure 4**), which indicated their roles as downstream components of *NPR1* during the AR response. On the other hand, the inductions of such downstream genes during the *P*. *syringae* DC3000-triggered AR were suppressed in the HvNPR1-Kd transgenic lines (**Figure 5**). Additionally, we used another reference gene, *HvActin* (GenBank accession number AK362208), to validate the results of our qRT-PCR assay for *HvPR1* and found similar expression patterns (**Supplementary Figure S3**).

### Differentially Expressed Genes (DEGs) Associated With *NPR1* Expression During AR

DEGs in different comparisons, including “WT_PST vs. WT_CK,” “OE_PST vs. OE_CK,” “OE_PST vs. WT_PST,” “Kd_PST vs. Kd_CK,” and “Kd_PST vs. WT_PST,” were identified by DESeq2 (*q*-value < 0.05 and | log2foldchange| > 1, with gene annotation). Three types of DEGs were manually classified based on their possible roles in the *NPR1*-mediated AR (**Supplementary Figure S4**). Type I DEGs were highly upregulated genes in the wNPR1-OE transgenic line after *P*. *syringae* DC3000 induction. A total of 24 Type I DEGs were designated from significantly upregulated genes based on the comparisons “OE_PST vs. OE_CK” and “OE_PST vs. WT_PST” (**Supplementary Figure S4** and **Table 1**). Several *PR* and *BCI* genes were annotated as Type I DEGs, including *probable glucan 1,3-beta-glucosidase A* (*HvPR2*), *peroxidase A2-like* (*HvPR9*), and *thionin BTH7-like* (*HvBCI7*). GO annotation for the Type I DEGs showed that the majority of these possible downstream genes of *NPR1* during AR were annotated with “binding” and “catalytic activity” in the molecular function category, and with “metabolic process” and “response to stimulus” in the biological process category (**Supplementary Figure S5A**).

**Table 1:** List of Type I DEGs.

Gene_id	WT_CK	WT_PST	OE_CK	OE_PST	Kd_CK	Kd_PST	Gene annotation information	log^2^ Fold Change OE_PSTvsOE_CK	padj	log^2^Fold Change OE_PSTvsWT_PST	padj
MLOC_65675	6.35	18.98	1.50	17.51	0.72	2.59	UDP-glycosyltransferase 74E1-like	1.91	2.42E-15	−0.02	0.97636
Novel05826	0.04	0.23	0.03	2.49	0.00	0.04	(3S,6E)-nerolidol synthase 1	1.85	9.01E-15	0.58	0.032943
MLOC_40202	0.37	0.44	0.44	36.86	0.09	0.20	Thionin BTH7-like	1.83	1.06E-14	0.61	0.014116
MLOC_13045	2.34	5.92	0.83	6.21	1.40	1.82	Multiprotein-bridging factor 1c	1.63	3.24E-11	0.04	0.95187
Novel05366	1.82	6.27	1.03	8.15	0.60	1.54	Extradiol ring-cleavage dioxygenase-like	1.60	1.32E-10	0.17	0.74561
MLOC_64594	0.72	2.98	0.68	8.25	0.68	0.99	Bowman-Birk type trypsin inhibitor-like	1.38	1.22E-07	0.42	0.24078
MLOC_56924	0.13	0.33	0.06	6.87	0.12	0.14	23 kDa jasmonate-induced protein-like	1.36	1.69E-08	0.47	0.060321
MLOC_81846	0.00	0.20	0.01	9.57	0.01	0.00	Subtilisin-chymotrypsin inhibitor-2A-like	1.36	1.05E-08	0.20	0.31317
Novel06256	0.00	1.07	0.35	6.66	0.00	0.12	Subtilisin inhibitor CLSI-I-like	1.21	3.01E-06	0.41	0.14977
MLOC_76394	1.20	5.14	1.60	6.11	0.41	0.63	Patatin-like protein 2	1.20	3.99E-06	0.14	0.80059
MLOC_60806	4.18	9.71	1.92	7.20	1.72	2.65	ABC transporter B family member 11-like	1.20	3.99E-06	−0.15	0.77755
Novel03795	8.30	17.72	5.15	17.08	5.56	8.00	Alcohol dehydrogenase 1	1.12	1.69E-05	0.00	0.99644
MLOC_22702	26.71	59.93	11.80	36.61	9.88	17.82	Flavonol 3-sulfotransferase-like	1.12	3.99E-06	−0.33	0.45406
MLOC_44543	18.33	42.15	17.45	43.89	18.65	38.20	Probable LRR receptor-like serine/threonine-protein kinase At3g47570	1.04	1.60E-07	0.09	0.85882
MLOC_2643	1.39	2.44	0.36	112.85	0.10	0.27	Subtilisin-chymotrypsin inhibitor-2A-like	1.03	1.98E-05	0.34	0.13263
Novel00269	69.17	106.80	26.96	74.38	31.19	44.56	Probable glucan 1,3-beta-glucosidase A	1.01	8.37E-05	−0.34	0.37308
MLOC_34615	0.08	0.03	0.03	3.51	0.00	0.02	Subtilisin-chymotrypsin inhibitor-2A-like	0.84	0.001085	1.16	NA
Novel06316	0.12	0.90	0.40	5.01	0.02	0.12	Enolase 1	0.64	0.045968	1.04	0.000726
MLOC_70788	0.86	0.59	0.67	2.58	0.36	0.61	Probable calcium-binding protein CML45	0.33	0.5337	1.09	0.000406
MLOC_39951	5.20	4.91	4.33	16.60	0.22	3.43	Tricetin 3′,4′,5′-O-trimethyltransferase-like	0.11	0.75237	1.11	0.000232
MLOC_79167	11.75	6.15	15.52	17.22	7.29	4.95	Peroxidase A2-like	0.02	0.9828	1.33	1.12E-11
MLOC_10292	2.19	0.91	2.62	2.37	1.99	0.85	F-box protein PP2-A13-like	−0.21	0.70551	1.17	2.60E-07
MLOC_15925	4.22	3.75	11.63	9.21	9.32	3.33	Cytochrome P450 86B1-like	−0.24	0.7385	1.03	0.000129
MLOC_56051	16.08	7.45	84.69	24.27	47.07	16.87	Chlorophyll a-b binding protein of LHCII type 1-like	−0.25	0.61004	1.15	8.38E-05

Type II DEGs were obtained and categorized into groups from significantly upregulated genes based on the comparisons “Kd_PST vs. Kd_CK” and “Kd_PST vs. WT_PST” (**Supplementary Figure S4** and **Supplementary Table S3**). A total of 64 genes were classified into this group, including several genes encoding transcription factors, such as *transcription factor JUNGBRUNNEN 1-like*, *probable WRKY transcription factor 48*, and *probable WRKY transcription factor 50*. Compared with GO annotations for the Type I DEGs, more genes in this group were annotated with “cellular process” but not “response to stimulus” (**Supplementary Figure S5B**). A third group of Type III DEGs, including only nine genes, were shared DEGs from Type I and Type II groups (**Supplementary Figures S4**, **S5C** and **Supplementary Table S4**).

### The Transient Expression of *HvWRKY6*, *HvWRKY40*, and *HvWRKY70* Enhanced Wheat Resistance to *Pt*

**FIGURE 6:**
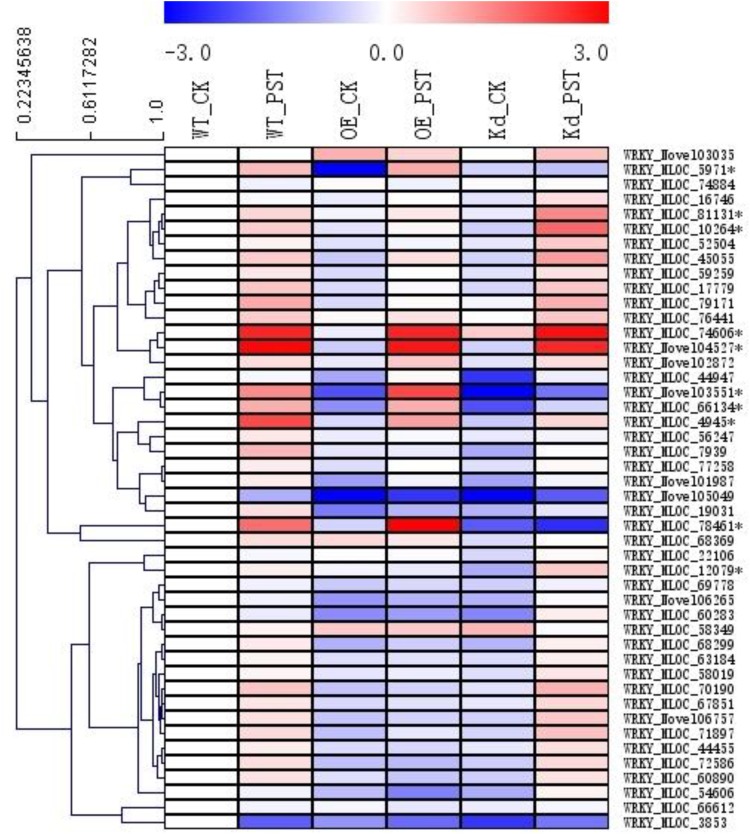
Expression patterns of *WRKY* transcription factor gene families during the *NPR1*-mediated AR. A heatmap was generated by MeV software using the FPKM values of the genes encoding WRKY transcription factors from the RNA-seq database. Ten differentially expressed *WRKYs* were selected (^∗^asterisk-labeled) for further functional characterization. Genes with similar expression patterns were clustered using the “Hierarchical Clustering” function of the MeV software.

A total of 46 *WRKY* genes were annotated in our RNA-seq database, and, based on their expression patterns, 10 of them were found to be involved in the *NPR1*-mediated AR (**Figure 6**). Since the naming system for the *WRKY* genes in the genera of *Triticeae* was confusing, a polygenetic tree was generated using sequences of all these 10 WRKY proteins and their homologs from *Hordeum vulgare* (Hv), *Aegilops tauschii* (At), *Brachypodium distachyon* (Bd), *Triticum aestivum* (Ta), *Triticum urartu* (Tu), and *Sorghum bicolor* (Sb) from the GenBank nr database (**Supplementary Figure S6**). All WRKYs were temporally designated according to their closest homologs in relative plant species, such as *HvWRKY4*, *HvWRKY6*, *HvWRKY17*, *HvWRKY19*, *HvWRKY20*, *HvWRKY31*, *HvWRKY40*, *HvWRKY64*, *HvWRKY70*, and *HvWRKY76*.

**FIGURE 7:**
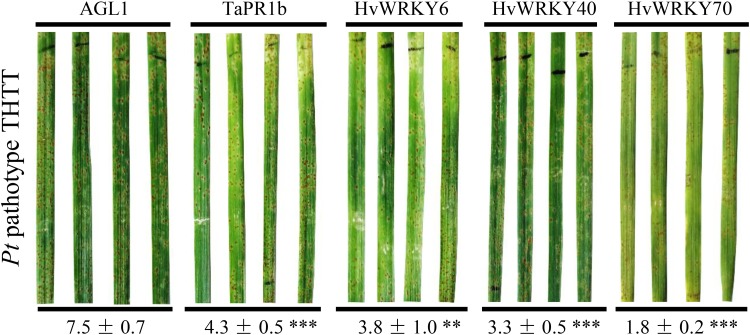
The transient expression of *HvWRKY6*, *HvWRKY40*, and *HvWRKY70*, enhanced the resistance of wheat to *Pt*. A total of 10 differentially expressed *WRKY* genes were transiently expressed in wheat leaves using *Agrobacterium*. Urediniospores of the highly virulent *Pt* pathotype THTT were spray-inoculated 4 days post-infiltration. Wheat transiently expressing *TaPR1b* was used as a positive control, whereas the untransformed *Agrobacterium* strain AGL1 was employed as a mock control. The phenotypes of leaf rust were recorded 10 days post-inoculation. The susceptible phenotypes were observed in all treatments. The numbers below the images of the leaves represent the average percentages of the *Pt* sporulation areas in the infiltration regions in each of the treatments. Asterisk indicates the significance of the differences between the treatment and mock established by using Dunnett’s test (^∗∗^*P* < 0.001, ^∗∗∗^*P* < 0.0001). The *Pt* sporulation in the wheat leaves transiently expressing *TaPR1b* and three *WRKYs*, including *HvWRKY6*, *HvWRKY40*, and *HvWRKY70*, was significantly reduced or delayed. Assay for each gene was performed at least twice and each repeat consisted of 5–18 biological replicates (**Supplementary Table S5**).

To test the potential of the differentially expressed *WRKYs* for improving wheat resistance to *Pt*, the open reading frame (ORF) of each *WRKY* gene was cloned and incorporated into a pLGY02 vector (Ubiquitin promoter, with T-DNA insertion site). The recombinant vector was transformed into the *Agrobacterium* strain AGL1. The transformed *Agrobacterium* was infiltrated into the secondary leaves of wheat seedlings of the susceptible line “Thatcher,” and the infiltration area was marked with a mark pen. Urediniospores of the highly virulent *Pt* pathotype THTT were spray-inoculated 4 days post-infiltration. The phenotype of leaf rust was recorded 10 days post-inoculation (**Figure 7**). The percentage of *Pt* sporulation area in the infiltration region of each leaf was calculated using ASSESS software. Wheat with transient expression of *TaPR1b* (GenBank accession number HQ541962) was utilized as a positive control, whereas the untransformed *Agrobacterium* strain AGL1 was employed as a negative control. Although susceptible phenotypes were observed in all treatments, *Pt* sporulation in the leaves of the wheat with transient expression of *TaPR1b* and three *WRKYs*, including *HvWRKY6*, *HvWRKY40*, and *HvWRKY70*, were significantly (*P* < 0.001) reduced or delayed (**Figure 7** and **Supplementary Table S5**). The transient expression of the other seven *WRKY* genes, including *HvWRKY4*, *HvWRKY17*, *HvWRKY19*, *HvWRKY20*, *HvWRKY31*, *HvWRKY64*, and *HvWRKY76*, in wheat leaves exerted no positive effect on wheat resistance to *Pt* (**Supplementary Table S5**).

## Discussion

*NPR1* has been reported as the master regulator of SAR in model plants of *Arabidopsis* and rice. Recent studies on *NPR1* homologs in wheat and barley have provided the initial clue to understand the mechanism of SAR in these two plant species (Cantu et al., 2013; Dey et al., 2014; Wang et al., 2016). Compared with SAR in *Arabidopsis*, three different SAR-like responses can be induced by either pathogens or SA/INA/BTH treatment in wheat and barley (Wang et al., 2018). In the current study, the observed beneficial effect of acquired resistance, which led to the reduction of *Pt* infection in wheat (**Figure 1**), suggested a potential use of *NPR1*-mediated AR in improving the resistance of *Triticeae* crops. The diminished AR to *M. oryzae* observed in the HvNPR1-Kd transgenic line indicated a key role of *NPR1* during the AR triggered by *P. syringae* DC3000 infection (**Figure 2**). Finally, the resistance of the wNPR1-OE transgenic line to *Mo* was enhanced (**Figure 2**), providing valuable evidence that *NPR1* can be utilized in improving barley resistance to *Mo*, possibly also to the recently emerged wheat blast disease (Inoue et al., 2017).

**FIGURE 8:**
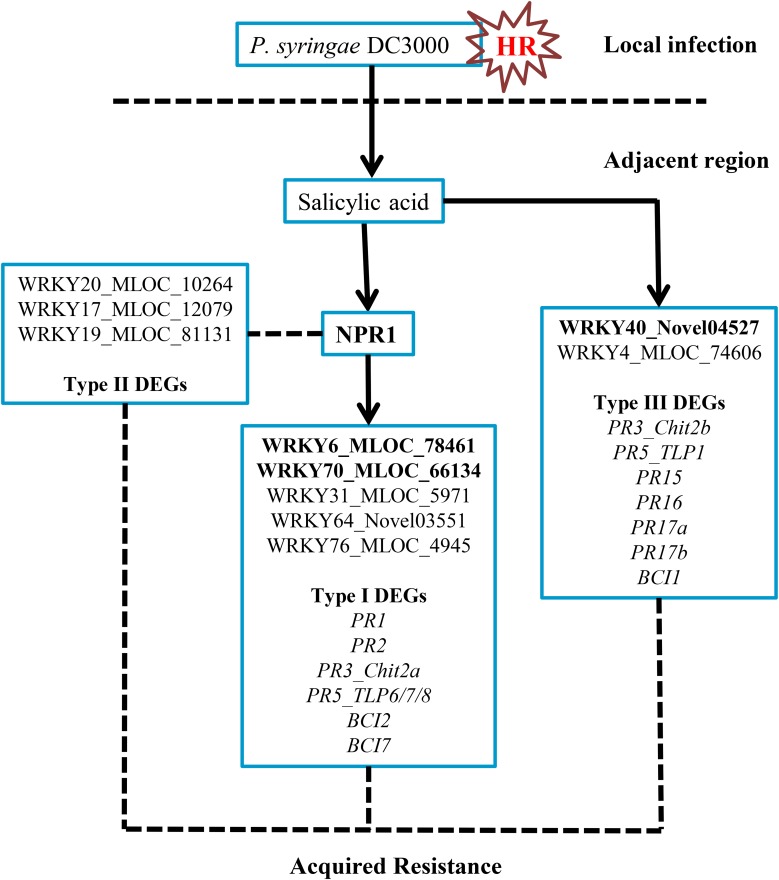
Possible regulatory network of the *NPR1*-mediated AR in barley. The transient expression of three barley *WRKY* genes, including *HvWRKY6*, *HvWRKY40*, and *HvWRKY70* (bold-labeled), in wheat leaves by *Agrobacterium*-mediated infiltration enhanced the resistance to *Pt*.

So far, a total number of 18 *PR* gene families have been designated from plant species, many of which showed involvement in wheat and barley resistance to various pathogens (Loon et al., 2006; Wang et al., 2018). Several barley *PR* genes, including *HvPR1*, *HvPR2*, *HvPR3_Chit2a*, *HvPR4b*, and *HvPR5_TLP6*, were validated as downstream genes of *NPR1* during the *P. syringae* DC3000-triggered AR (Wang et al., 2016). Another group of *BCI* genes seems to be responsible for the enhanced resistance in barley induced by BTH treatment (Beßer et al., 2000). In the present study, the expression profiles of all the *PR* and *BCI* genes were generated using FPKM values from the RNA-seq assay. The *NPR1*-regulated genes, including *HvPR1*, *HvPR2*, *HvPR3_Chit2a*, *HvPR5_TLP6/7/8*, *HvPR13/BCI2*, and *HvBCI7*, during the *P. syringae* DC3000-triggered AR, were established (**Figures 3**, **8**). Further studies on *HvPR13/BCI2* and *HvBCI7* may provide initial evidence for understanding the relationship between AR and BIR. Interestingly, the expression levels of several *PR* genes, including *HvPR3_Chit2b*, *HvPR5_TLP1*, *HvPR15*, *HvPR16*, *HvPR17a*, and *HvPR17b*, were induced in a *NPR1*-independent manner, which indicated that other unknown regulators were functioning during the AR triggered by *P. syringae* DC3000.

In this investigation, we classified three DEG groups based on their expression patterns. Several *PR* and *BCI* genes were categorized into Type I and Type III DEGs (**Table 1** and **Supplementary Table S4**). The possible downstream genes of *NPR1* during the *P. syringae* DC3000-triggered AR were mainly identified as Type I DEGs. Interestingly, we also discovered a large group of Type II DEGs (**Supplementary Table S3**) which were highly induced only in the HvNPR1-Kd transgenic line after *P. syringae* DC3000 infection. We speculated that some of them were negative regulators or upstream genes of *NPR1* during the AR. Furthermore, the regulator network of *NPR1* in barley during the *P. syringae* DC3000-triggered AR was initially established in our investigation (**Figure 8**).

In addition, we checked the involvement of barley *WRKY* transcription factors in the *NPR1*-mediated AR. In model plants of *Arabidopsis* and rice, several *WRKYs*, including *AtWRKY18*, *AtWRKY58*, *AtWRKY70*, *OsWRKY03*, *OsWRKY71*, and *OsWRKY45*, have been suggested to play important roles in the SAR (Liu et al., 2005, 2007; Wang et al., 2006; Shimono et al., 2007; Nakayama et al., 2013). A total of 171 *TaWRKYs* were identified in wheat, whereas 45 *HvWRKYs* were detected in barley (Christer et al., 2008; Pan et al., 2017). It was rather difficult to determine the key *WRKY* genes during SAR in these two crop species. By checking the expression patterns of all the 46 *WRKYs* identified in our RNA-seq assay (**Figure 6**), a total of 10 *HvWRKY* genes with relatively higher induction in either the wNPR1-OE or HvNPR1-Kd transgenic line were selected for further functional validation.

Three differentially expressed *HvWRKYs*, including *HvWRKY6*, *HvWRKY40*, and *HvWRKY70*, exerted positive effects on wheat resistance to *Pt* (**Figure 7**). HvWRKY6_MLOC_78461 was a barley homolog of AtWRKY6_XP_020181741 (**Supplementary Figure S6**), which had been reported to be associated with both senescence- and defense-related processes (Robatzek and Somssich, 2001). HvWRKY40_NOVEL04527 was clustered with AtWRKY40_EMT21551 in our polygenetic analysis (**Supplementary Figure S6**). In an earlier study, AtWRKY40 was found to be a repressor of antimycin A-induced mitochondrial retrograde expression and high-light-induced signaling (Aken et al., 2013). In addition, our data evidenced that HvWRKY70_MLOC_66134 was clustered with *Arabidopsis* AtWRKY70_XP_020165252 (**Supplementary Figure S6**), which exerted dual roles as negative regulators of SA biosynthesis and positive regulators of SA-mediated gene expression and resistance in *Arabidopsis* (Wang et al., 2006). We speculated that these three *HvWRKYs* were key regulators during the *NPR1*-mediated AR and may represent valuable transgenic resources for resistance improvement of wheat plants.

## Conclusion

In conclusion, *NPR1* acts as a key regulator during the *P. syringae* DC3000-triggered AR in barley (**Figure 8**). In the present study, we have identified genes associated with the *NPR1*-mediated AR. Several *PR* and *BCI* genes were confirmed to be the downstream genes of *NPR1*. The expression levels of several *WRKY* transcription factors were significantly associated with *NPR1* expression, which might be key components in the *NPR1*-mediated AR. Furthermore, it is noteworthy that three differentially expressed *WRKYs* identified in the current study showed potential for resistance improvement of wheat plants.

## Author Contributions

XW conceived the original screening and research plans. XW and DL supervised the experiments. JG and WB performed most of the experiments. HL, JW, and XY provided technical assistance to JG. XW designed the experiments and analyzed the data. XW conceived the project and wrote the article with contributions of all the authors. DL supervised and complemented the writing.

## Conflict of Interest Statement

The authors declare that the research was conducted in the absence of any commercial or financial relationships that could be construed as a potential conflict of interest.
