# Supplementary material for: WRKY Transcription Factors Associated With NPR1-Mediated Acquired Resistance in Barley Are Potential Resources to Improve Wheat Resistance to Puccinia triticina
Source: Front Plant Sci. 2018 Oct 17;9:1486. doi: 10.3389/fpls.2018.01486 (PMC6199750; doi:10.3389/fpls.2018.01486)
Supplement: Supplementary file 1 [file Image_1.pdf]

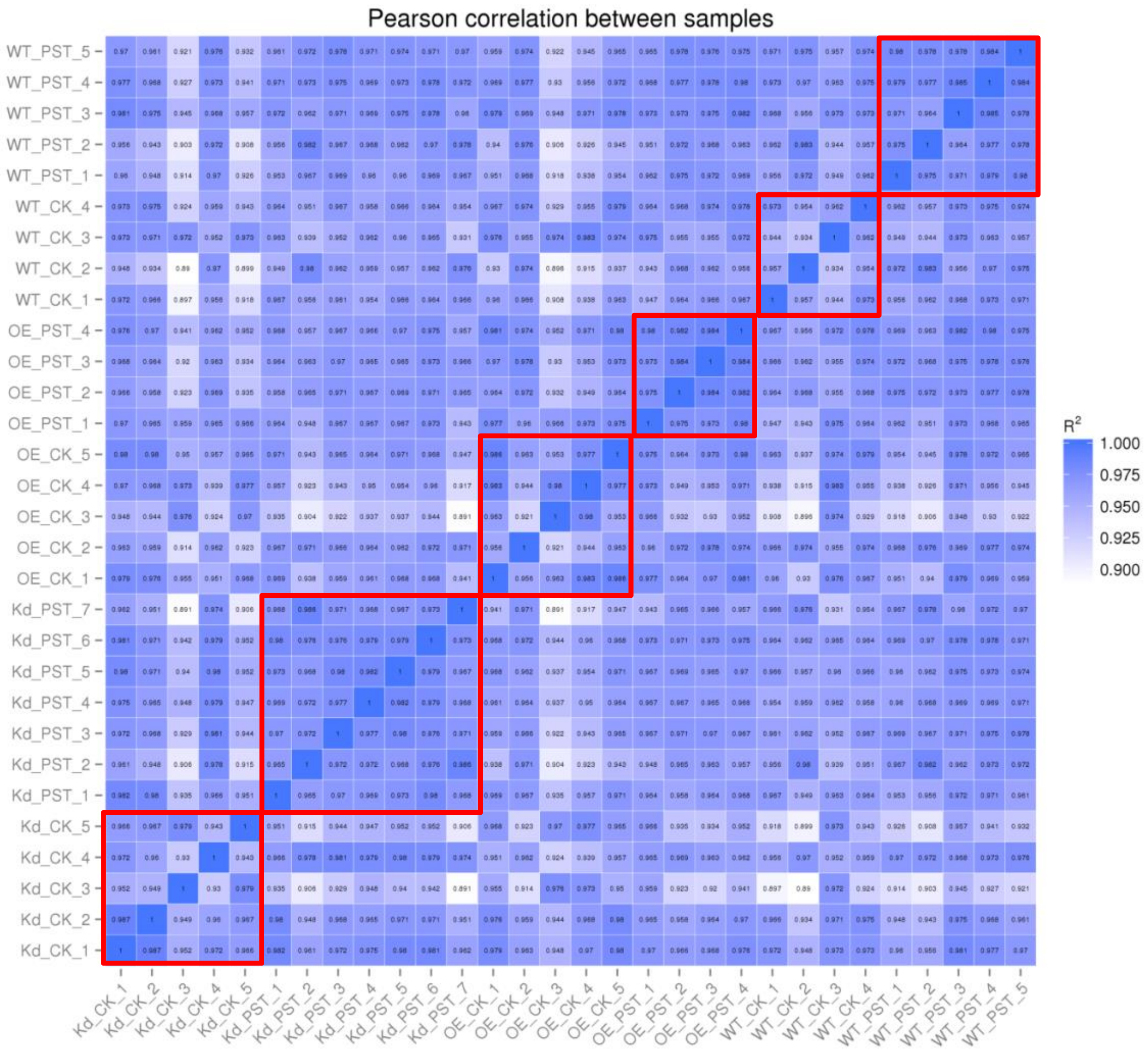

Supplementary Figure S1. Pearson's correlation of gene expression levels between biological replicates in the present RNA-seq assay. A number of 4-7 biological replicates of each treatment and genotype combination were included in the analysis. A clear correlation of gene expression level between biological replicates was observed ($R^2 > 0.92$). CK, water infiltration control; PST, *P. syringae* DC3000 infiltration; OE, wNPR1-OE transgenic line; Kd, HvNPR1-Kd transgenic line.
