# Supplementary material for: WRKY Transcription Factors Associated With NPR1-Mediated Acquired Resistance in Barley Are Potential Resources to Improve Wheat Resistance to Puccinia triticina
Source: Front Plant Sci. 2018 Oct 17;9:1486. doi: 10.3389/fpls.2018.01486 (PMC6199750; doi:10.3389/fpls.2018.01486)
Supplement: Supplementary file 2 [file Image_2.pdf]

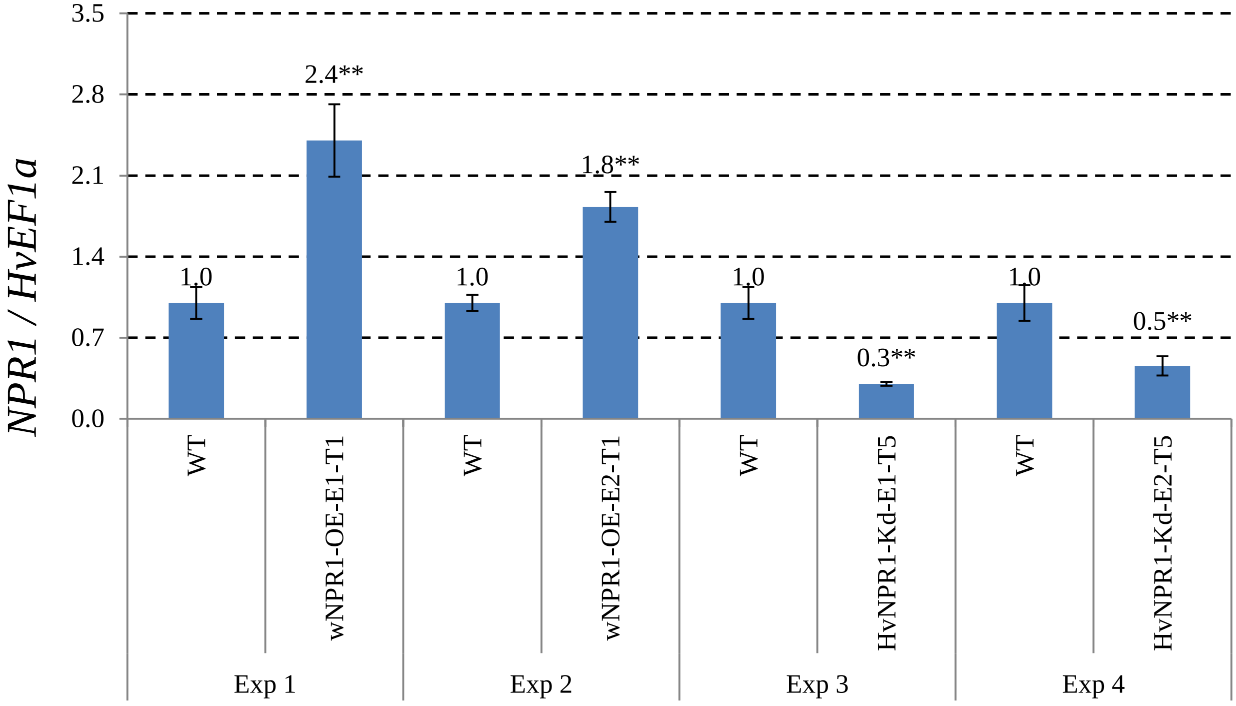

Supplementary Figure S2. Transcription levels of *NPR1* in the barley transgenic lines wNPR1-OE and HvNPR1-Kd and the wild-type plants measured by qRT-PCR. Transcript levels are expressed relative to those of the endogenous control *HvEF1a* using the $2^{-\triangle CT}$ method. Each experiment, consisting of 4-11 biological replicates, was considered as a block. Calculations of the mean and standard error were performed using Microsoft Excel software. Data were transformed to restore normality, and two-sample t-test (** $P < 0.01$) was conducted using SAS software version 9.4.
