# Supplementary material for: WRKY Transcription Factors Associated With NPR1-Mediated Acquired Resistance in Barley Are Potential Resources to Improve Wheat Resistance to Puccinia triticina
Source: Front Plant Sci. 2018 Oct 17;9:1486. doi: 10.3389/fpls.2018.01486 (PMC6199750; doi:10.3389/fpls.2018.01486)
Supplement: Supplementary file 3 [file Image_3.pdf]

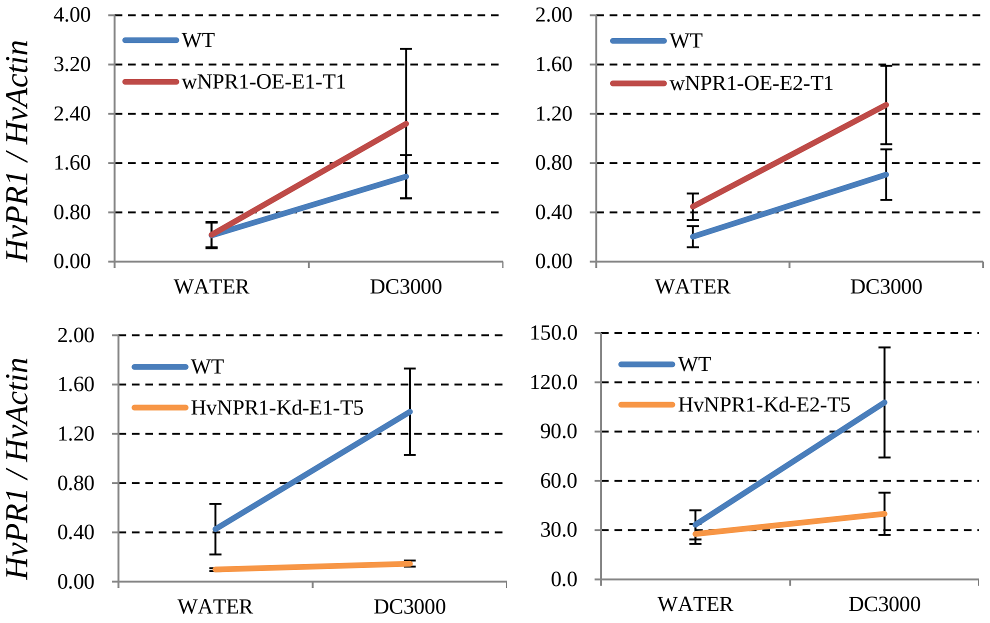

Combined *P* value:
WT: DC3000 vs WATER = **0.0341***
WATER: OE vs WT = 0.3590
DC3000: OE vs WT = **0.0406***

Combined *P* value:
WATER: Kd vs WT = 0.1991
DC3000: Kd vs WT = **0.0342***

Supplementary Figure S3. Validation of the expression levels of *HvPR1* gene by qRT-PCR using *HvActin* as the reference gene. Third leaves of the wNPR1-OE and HvNPR1-Kd barley transgenic lines and wild-type plants were infiltrated with water (control) or *Pseudomonas syringae* pv. *tomato* DC3000. Samples for qRT-PCR assays were collected from the leaf region adjacent to the infection 48 hours after inoculation, after a cell death phenotype observed. The transcript levels are expressed relative to those of the endogenous control *HvActin* using the $2^{-\triangle CT}$ method. Two independent transgenic lines for each of the wNPR1-OE and HvNPR1-Kd were used. Each experiment, consisting of 4-11 biological replicates, was considered as a block. Calculations of the mean and standard error were performed using Microsoft Excel software. Data were transformed to restore normality, and general linearized model (GLM) ANOVA (* $P < 0.05$) was conducted using SAS software version 9.4.
