# Supplementary material for: WRKY Transcription Factors Associated With NPR1-Mediated Acquired Resistance in Barley Are Potential Resources to Improve Wheat Resistance to Puccinia triticina
Source: Front Plant Sci. 2018 Oct 17;9:1486. doi: 10.3389/fpls.2018.01486 (PMC6199750; doi:10.3389/fpls.2018.01486)
Supplement: Supplementary file 4 [file Image_4.pdf]

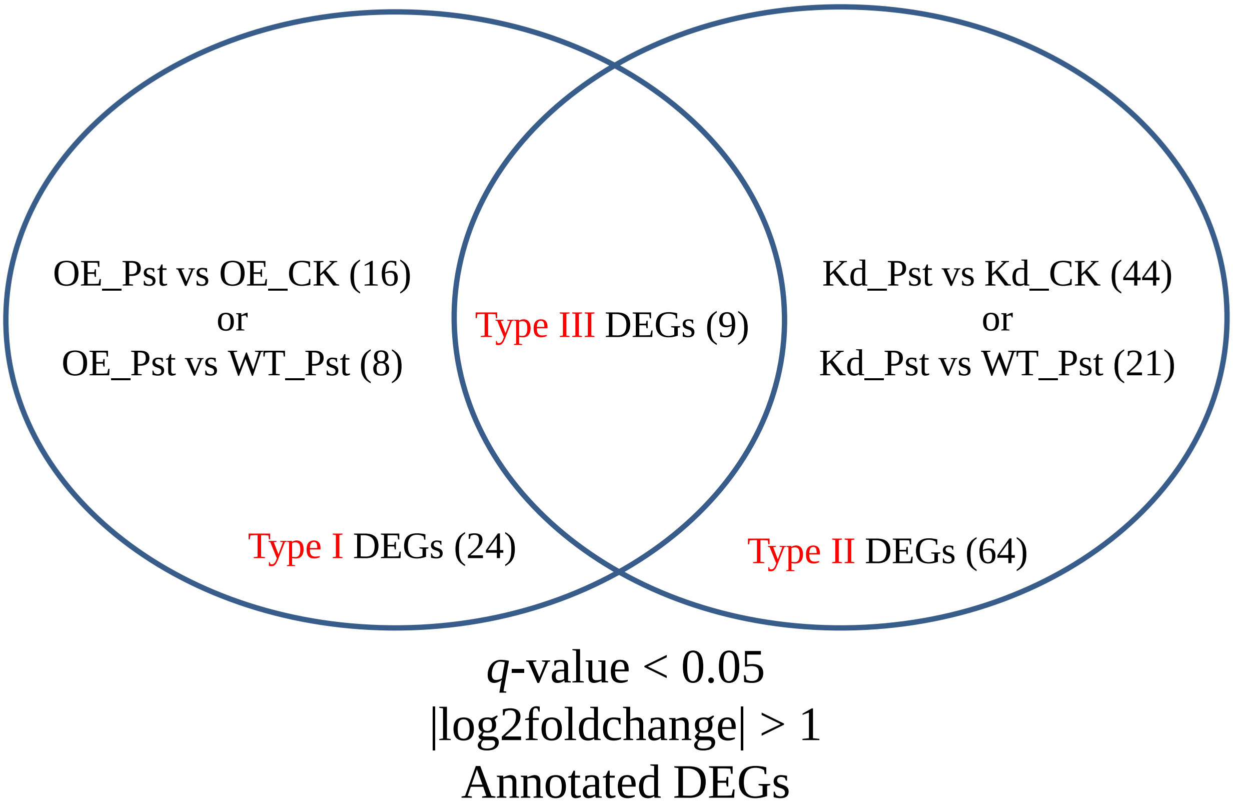

Supplementary Figure S4. Three types of DEGs were manually classified based on their possible roles in the *NPR1*-mediated AR. DEGs were identified by DESeq2 (q-value < 0.05 and |log2foldchange| > 1, with gene annotation). Type I DEGs were designated from significantly upregulated genes from comparisons “OE_PST vs OE_CK” and “OE_PST vs WT_PST”. Type II DEGs were designated from significantly upregulated genes from comparisons “Kd_PST vs Kd_CK” and “Kd_PST vs WT_PST”. A third group of Type III DEGs were shared DEGs from Type I and Type II groups. CK, water infiltration control; PST, *P. syringae* DC3000 infiltration; OE, wNPR1-OE transgenic line; Kd, HvNPR1-Kd transgenic line.
