# Supplementary material for: WRKY Transcription Factors Associated With NPR1-Mediated Acquired Resistance in Barley Are Potential Resources to Improve Wheat Resistance to Puccinia triticina
Source: Front Plant Sci. 2018 Oct 17;9:1486. doi: 10.3389/fpls.2018.01486 (PMC6199750; doi:10.3389/fpls.2018.01486)
Supplement: Supplementary file 5 [file Image_5.pdf]

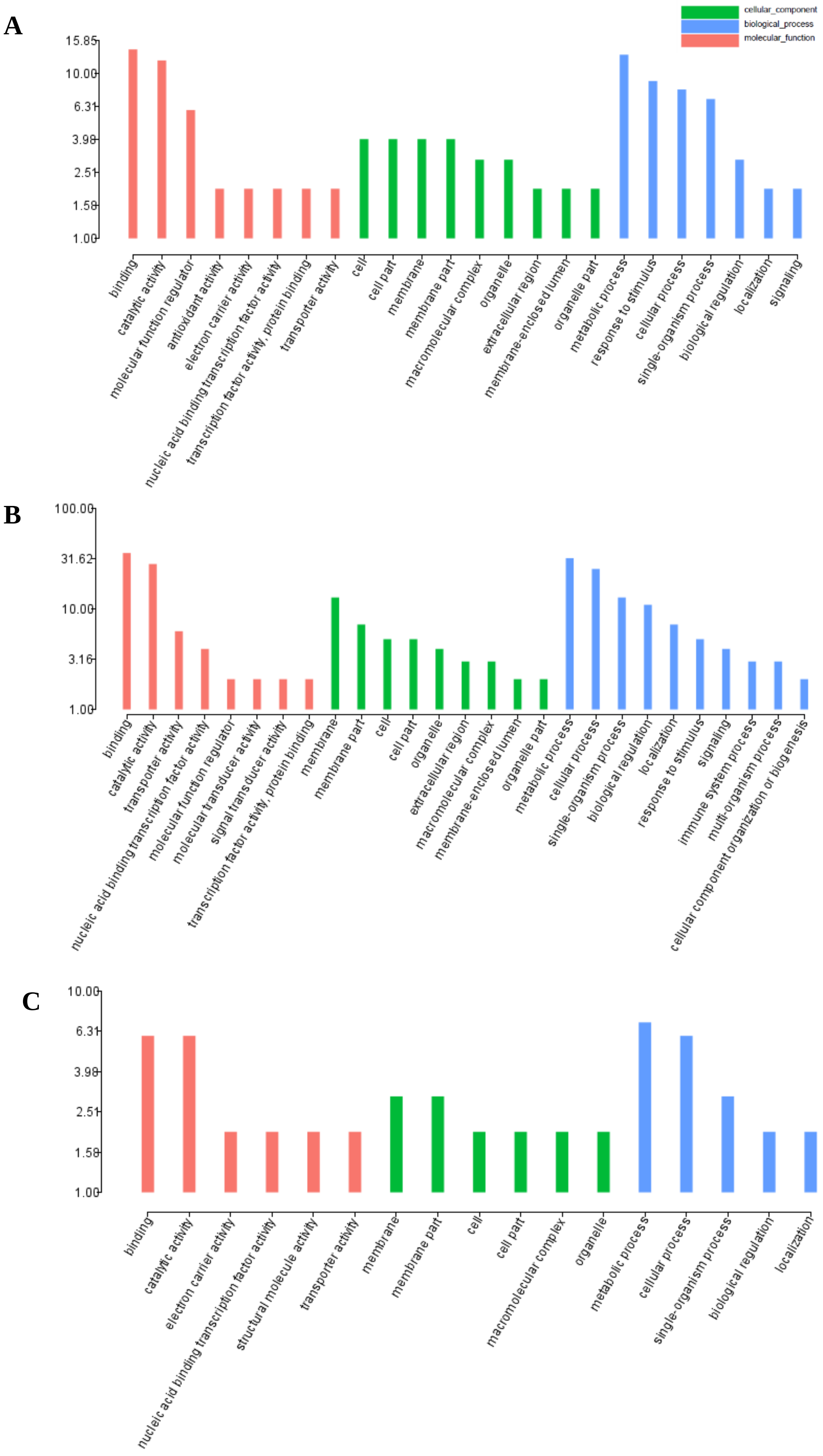

Supplementary Figure S5. GO annotation for DEGs. All the Type I **(A)**, Type II **(B)**, and Type III **(C)** DEGs were categorized by their GO annotations into different functional groups of three main categories: biological process, cellular component, and molecular function. The y-axis indicates the percentage of genes in a category.
