# Supplementary material for: WRKY Transcription Factors Associated With NPR1-Mediated Acquired Resistance in Barley Are Potential Resources to Improve Wheat Resistance to Puccinia triticina
Source: Front Plant Sci. 2018 Oct 17;9:1486. doi: 10.3389/fpls.2018.01486 (PMC6199750; doi:10.3389/fpls.2018.01486)
Supplement: Supplementary file 6 [file Image_6.pdf]

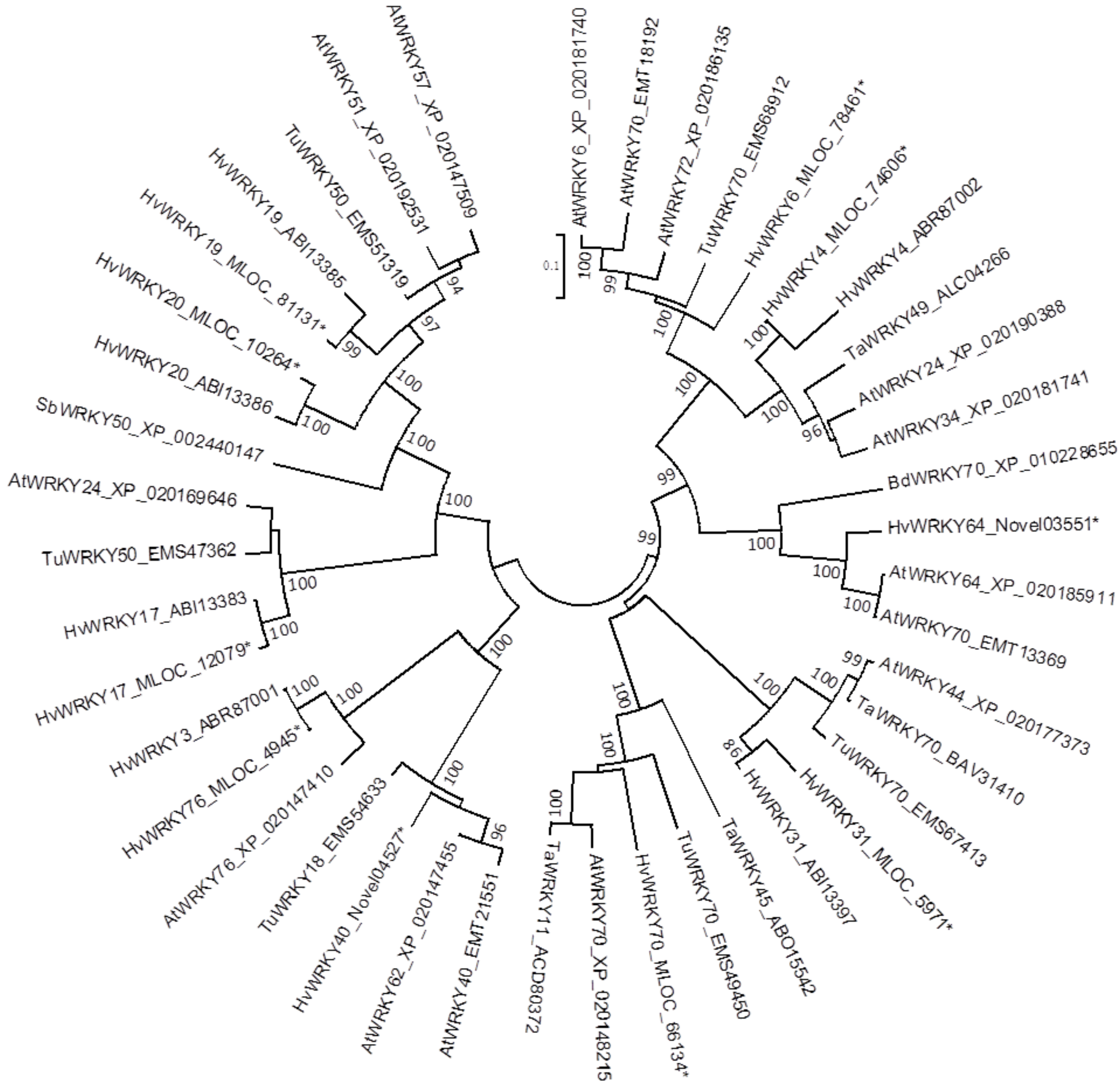

Supplementary Figure S6. The polygenetic tree of the characterized WRKY proteins in the RNA-seq database. A neighbor joining tree was generated by MEGA software using all of the ten WRKY proteins selected from our RNA-seq database (* star labeled) and their homologs from *Hordeum vulgare* (Hv), *Aegilops tauschii* (At), *Brachypodium distachyon* (Bd), *Triticum aestivum* (Ta), *Triticum urartu* (Tu), and *Sorghum bicolor* (Sb) obtained from GenBank nr database. All the WRKYs were temporally designated according to their closest homologs.
