# Supplementary material for: WRKY Transcription Factors Associated With NPR1-Mediated Acquired Resistance in Barley Are Potential Resources to Improve Wheat Resistance to Puccinia triticina
Source: Front Plant Sci. 2018 Oct 17;9:1486. doi: 10.3389/fpls.2018.01486 (PMC6199750; doi:10.3389/fpls.2018.01486)
Supplement: Supplementary file 7 [file Table_1.docx]

**Supplementary Table S1.** Primers used in this study.

| **Primer usage** | **Primer Name** | **Primer sequences 5' to 3'** | **PCR product** | **Accession** | **Efficiency** |
| --- | --- | --- | --- | --- | --- |
| qRT-PCR assay | HvPR1b-qRT-F | CCAAGCTAGCCATCTTGCTC | 196 bp | X74940 | 85.0% |
|  | HvPR1b-qRT-R | TTGCAGTCGTTGATCCTCTG |  |  |  |
|  | HvPR2-qRT-F | AAGATGTTGCCTCCATGTTTGCAG | 175 bp | M62907 | 96.7% |
|  | HvPR2-qRT-R | AAGTAGATGCGCATGCCGTTGAT |  |  |  |
|  | HvPR3_Chit2a-qRT-F | GGTTCCAGGCTACGGTGTAA | 163 bp | X78671 | 100.0% |
|  | HvPR3_Chit2a-qRT-R | GTTCCGTTGGGTGTAGCAGT |  |  |  |
|  | HvPR5_TLP6-qRT-F | CAAGAGCGGTATCATCCATCC | 198 bp | AF355456 | 93.1% |
|  | HvPR5_TLP6-qRT-R | CATGTTCAGCGCCCACGA |  |  |  |
|  | HvEF1a-qRT-F | TGGTGTCATCAAGCCTGGTATGGT | 86 bp | Z50789 | 100.1% |
|  | HvEF1a-qRT-R | ACTCATGGTGCATCTCAACGGACT |  |  |  |
|  | HvActin-qRT-F | AAGTACAGTGTCTGGATTGGAGGG | 130 bp | AK362208.1 | 104.9% |
|  | HvActin-qRT-R | TCGCAACTTAGAAGCACTTCCG |  |  |  |
|  | HvBCI1-qRT-F | GGGACAAGTTTGCTTGGTTCAG | 116 bp | U56406 | 99.3% |
|  | HvBCI1-qRT-R | CTCGTCCAGATTGCTCTTGATAGG |  |  |  |
|  | HvBCI3-qRT-F | GGCCGGACGGAGGATAAGAG | 185 bp | AJ250282 | 89.4% |
|  | HvBCI3-qRT-R | CACTGGTCGCCGATGTTGC |  |  |  |
|  | HvBCI6-qRT-F | CACGACGGACAGGCTGATTG | 292 bp | CAB71339 | 97.3% |
|  | HvBCI6-qRT-R | GTCCACCTTGTACCAGAAGTTTGC |  |  |  |
|  | HvBCI7-qRT-F | CGGAGGTAGCGGGAAAGTC | 123 bp | AJ250663 | 98.3% |
|  | HvBCI7-qRT-R | CACGGTTGGTCCTGAAGTCG |  |  |  |
|  | NPR1-qRT-F | CCAAAACAGTCGAACTCGGCAA | 217 bp | JX424315 | 94.7% |
|  | NPR1-qRT-R | GACGATGAGGAAGATGAAAGGGTTG |  |  |  |
| Transient expression assay | TaPR1b-ORF-F | GGTACCATGGAGACGCCCAAGCTGGC | 495 bp | HQ541962 |  |
|  | TaPR1b-ORF-R | ACTAGTTTAGTATGGTTTCTGTCCAATGACATTC |  |  |  |
|  | HvWRKY70-ORF-F | GGTACCATGGAGACCCCGTTCGCG | 924 bp | MLOC_66134 |  |
|  | HvWRKY70-ORF-R | ACTAGTTCAATGATCGAGTACGTACGACGC |  |  |  |
|  | HvWRKY6-ORF-F | GGTACCATGGCGTCTTCCGGCGG | 1023 bp | MLOC_78461 |  |
|  | HvWRKY6-ORF-R | ACTAGTCTAGATTCGCCAGTGGCAAACG |  |  |  |
|  | HvWRKY19-ORF-F | GGTACCATGGCGGCAGTCGGAGCG | 657 bp | MLOC_81131 |  |
|  | HvWRKY19-ORF-R | ACTAGTTCAGTTGAGGGAGCCTGGCG |  |  |  |
|  | HvWRKY40-ORF-F | GGATCCATGGACGACGGTTCGTCATGT | 810 bp | Novel04527 |  |
|  | HvWRKY40-ORF-R | ACTAGTTTAACTTAGCCGGGCTGGAATAA |  |  |  |
|  | HvWRKY20-ORF-F | GGTACCATGGCGATAGTCGGAGCG | 651 bp | MLOC_10264 |  |
|  | HvWRKY20-ORF-R | ACTAGTCTAATTGAGTGAGCCTGGTGG |  |  |  |
|  | HvWRKY76-ORF-F | GGTACCATGGAAACGGCGCGGTG | 996 bp | MLOC_4945 |  |
|  | HvWRKY76-ORF-R | ACTAGTCTAATAATCCGGCAGCTTCCG |  |  |  |
|  | HvWRKY4-ORF-F | GGTACCATGGCATCTTGTGGCGG | 1047 bp | MLOC_74606 |  |
|  | HvWRKY4-ORF-R | ACTAGTTCAGGAATCGAAGCCGAA |  |  |  |
|  | HvWRKY17-ORF-F | GGTACCATGGCGGCTTCGTTGGGAC | 600 bp | MLOC_12079 |  |
|  | HvWRKY17-ORF-R | ACTAGTTCAGAATTGCAGTAGGCCAGCG |  |  |  |
|  | HvWRKY31-ORF-F | GGTACCATGGCGGCACTTGTCACT | 930 bp | MLOC_5971 |  |
|  | HvWRKY31-ORF-R | ACTAGTCTATTGGAACAGAGGAATGGC |  |  |  |
|  | HvWRKY64-ORF-F | GGTACCATGGCATCTTCGGACAGCG | 897 bp | Novel03551 |  |
|  | HvWRKY64-ORF-R | ACTAGTTCATGGATCGAACTGGAAGAAAT |  |  |  |
