# Supplementary material for: WRKY Transcription Factors Associated With NPR1-Mediated Acquired Resistance in Barley Are Potential Resources to Improve Wheat Resistance to Puccinia triticina
Source: Front Plant Sci. 2018 Oct 17;9:1486. doi: 10.3389/fpls.2018.01486 (PMC6199750; doi:10.3389/fpls.2018.01486)
Supplement: Supplementary file 8 [file Table_2.docx]

**Supplementary Table S2.** Sequencing information of the samples in the RNA-seq assay.

| **Sample name** | **Raw reads** | **Clean reads** | **Clean bases (Gb)** | **Error rate (%)** | **Q20(%)** | **Q30(%)** | **GC content**  **(%)** |
| --- | --- | --- | --- | --- | --- | --- | --- |
| WT_CK_1 | 47319652 | 44551294 | 6.68 | 0.02 | 96.58 | 91.58 | 55.68 |
| WT_CK_2 | 45519898 | 42764160 | 6.41 | 0.02 | 96.58 | 91.62 | 56.39 |
| WT_CK_3 | 58925588 | 55559346 | 8.33 | 0.02 | 96.25 | 90.93 | 57.39 |
| WT_CK_4 | 43630178 | 41130726 | 6.17 | 0.02 | 96.25 | 90.96 | 56.77 |
| WT_PST_1 | 44160086 | 42561596 | 6.38 | 0.02 | 94.62 | 87.46 | 57.3 |
| WT_PST_2 | 57389802 | 53333296 | 8.00 | 0.02 | 96.96 | 92.3 | 52.68 |
| WT_PST_3 | 47878446 | 46158266 | 6.92 | 0.02 | 95.01 | 88.2 | 57.06 |
| WT_PST_4 | 53544932 | 45815184 | 6.87 | 0.02 | 96.19 | 89.91 | 57.17 |
| WT_PST_5 | 53420640 | 51490486 | 7.72 | 0.02 | 95.27 | 88.8 | 56.99 |
| Kd_CK_1 | 53055020 | 49968812 | 7.50 | 0.02 | 96.44 | 91.29 | 56.7 |
| Kd_CK_2 | 60185200 | 57818786 | 8.67 | 0.02 | 95.92 | 90.12 | 57.08 |
| Kd_CK_3 | 49888624 | 47028112 | 7.05 | 0.02 | 96.06 | 90.53 | 57.57 |
| Kd_CK_4 | 45571894 | 42795016 | 6.42 | 0.02 | 96.56 | 91.51 | 56.27 |
| Kd_CK_5 | 45070172 | 42458826 | 6.37 | 0.02 | 96.4 | 91.18 | 57.44 |
| Kd_PST_1 | 46280462 | 44528526 | 6.68 | 0.02 | 96.34 | 90.88 | 56.24 |
| Kd_PST_2 | 44808904 | 43209844 | 6.48 | 0.02 | 95.94 | 90.08 | 56.13 |
| Kd_PST_3 | 42552242 | 41069044 | 6.16 | 0.02 | 96.11 | 90.39 | 56.22 |
| Kd_PST_4 | 48045244 | 46230758 | 6.93 | 0.02 | 95.91 | 89.92 | 56.12 |
| Kd_PST_5 | 45925168 | 44296048 | 6.64 | 0.02 | 96.06 | 90.37 | 55.42 |
| Kd_PST_6 | 50042174 | 48393762 | 7.26 | 0.02 | 96.36 | 90.86 | 55.96 |
| Kd_PST_7 | 46405956 | 44851460 | 6.73 | 0.02 | 96.45 | 91.1 | 55.95 |
| OE_CK_1 | 57490684 | 55470360 | 8.32 | 0.02 | 96 | 90.12 | 56.15 |
| OE_CK_2 | 47606252 | 45867648 | 6.88 | 0.02 | 96.42 | 91.02 | 56.45 |
| OE_CK_3 | 48362682 | 46784994 | 7.02 | 0.02 | 96.41 | 90.96 | 56.4 |
| OE_CK_4 | 47317396 | 45668702 | 6.85 | 0.02 | 96.11 | 90.38 | 56.79 |
| OE_CK_5 | 48368996 | 46507626 | 6.98 | 0.02 | 96.32 | 90.82 | 56.46 |
| OE_PST_1 | 45960266 | 44248598 | 6.64 | 0.02 | 96.41 | 91 | 56.14 |
| OE_PST_2 | 46817858 | 45019590 | 6.75 | 0.02 | 96.35 | 90.9 | 55.99 |
| OE_PST_3 | 46536174 | 44999130 | 6.75 | 0.02 | 96.46 | 91.08 | 55.95 |
| OE_PST_4 | 48999226 | 47257122 | 7.09 | 0.02 | 95.91 | 89.96 | 55.9 |

CK, water infiltration control; PST, *P*. *syringae* DC3000 infiltration; OE, wNPR1-OE transgenic line; Kd, HvNPR1-Kd transgenic line.
