# Supplementary material for: WRKY Transcription Factors Associated With NPR1-Mediated Acquired Resistance in Barley Are Potential Resources to Improve Wheat Resistance to Puccinia triticina
Source: Front Plant Sci. 2018 Oct 17;9:1486. doi: 10.3389/fpls.2018.01486 (PMC6199750; doi:10.3389/fpls.2018.01486)
Supplement: Supplementary file 9 [file Table_3.docx]

**Supplementary Table S3.** List of Type II DEGs.

| **Gene_id** | **WT_CK** | **WT_PST** | **OE_CK** | **OE_PST** | **Kd_CK** | **Kd_PST** | **Gene Annotation Information** | **log2FoldChange Kd_PSTvsKd_CK** | **padj** | **log2FoldChange Kd_PSTvsWT_PST** | **padj** |
| --- | --- | --- | --- | --- | --- | --- | --- | --- | --- | --- | --- |
| MLOC_23887 | 1.01 | 1.60 | 0.86 | 1.66 | 0.28 | 1.52 | RING-H2 finger protein ATL64-like | 1.69 | 1.30E-07 | -0.04 | 0.95046 |
| MLOC_72673 | 0.54 | 0.92 | 0.40 | 1.01 | 0.22 | 1.59 | lipid phosphate phosphatase 2-like | 1.59 | 8.49E-05 | 0.39 | 0.33452 |
| MLOC_53505 | 1.87 | 3.91 | 1.68 | 3.07 | 0.46 | 2.41 | 7-deoxyloganetin glucosyltransferase-like | 1.54 | 4.56E-05 | -0.29 | 0.52038 |
| MLOC_66348 | 0.26 | 0.38 | 0.26 | 0.41 | 0.10 | 0.52 | transcription factor JUNGBRUNNEN 1-like | 1.39 | 0.00088435 | 0.22 | 0.65772 |
| MLOC_54176 | 0.23 | 0.32 | 0.14 | 0.15 | 0.07 | 0.37 | synaptonemal complex protein 2-like | 1.36 | 0.00088739 | 0.11 | 0.8613 |
| MLOC_10264 | 4.21 | 6.40 | 3.35 | 4.49 | 2.83 | 14.63 | probable WRKY transcription factor 48 | 1.35 | 0.0014269 | 0.61 | 0.059554 |
| MLOC_68184 | 29.63 | 59.52 | 31.59 | 88.35 | 12.57 | 60.24 | chitinase 8-like | 1.35 | 0.0012355 | 0.01 | 0.98937 |
| MLOC_81131 | 8.14 | 11.51 | 7.50 | 9.93 | 6.85 | 21.47 | probable WRKY transcription factor 48 | 1.28 | 1.71E-06 | 0.60 | 0.047278 |
| MLOC_63061 | 0.62 | 0.87 | 0.42 | 0.51 | 0.19 | 0.88 | BTB/POZ and MATH domain-containing protein 1-like | 1.25 | 0.0035123 | 0.01 | 0.99097 |
| MLOC_66939 | 1.14 | 2.26 | 1.03 | 0.73 | 0.22 | 1.08 | protein NRT1/ PTR FAMILY 7.2-like | 1.25 | 0.0046051 | -0.49 | 0.16593 |
| MLOC_53526 | 0.72 | 1.18 | 0.55 | 0.59 | 0.32 | 1.28 | zinc finger protein STAR3-like | 1.24 | 0.0020955 | 0.06 | 0.93288 |
| MLOC_24900 | 0.31 | 0.57 | 0.97 | 0.29 | 0.08 | 0.55 | L-ascorbate oxidase-like | 1.24 | 0.0063466 | -0.01 | 0.99039 |
| MLOC_12079 | 7.71 | 8.78 | 7.12 | 6.56 | 3.93 | 11.96 | probable WRKY transcription factor 50 | 1.23 | 1.64E-05 | 0.29 | 0.50873 |
| MLOC_51955 | 0.53 | 0.65 | 0.86 | 0.41 | 0.17 | 0.83 | aspartic proteinase nepenthesin-1-like | 1.23 | 0.0057475 | 0.14 | 0.80219 |
| MLOC_78131 | 0.86 | 0.94 | 0.33 | 0.32 | 1.42 | 4.91 | probable LRR receptor-like serine/threonine-protein kinase At4g08850 | 1.22 | 0.0012834 | 1.80 | 3.57E-21 |
| MLOC_74471 | 2.77 | 3.52 | 1.86 | 2.75 | 1.45 | 5.10 | putative disease resistance protein At4g10780 | 1.21 | 0.0017092 | 0.23 | 0.64078 |
| MLOC_13737 | 1.42 | 2.22 | 0.94 | 1.90 | 1.18 | 4.06 | probable LRR receptor-like serine/threonine-protein kinase At4g08850 | 1.19 | 0.0026723 | 0.59 | 0.056877 |
| Novel02151 | 0.45 | 0.53 | 0.30 | 0.18 | 0.14 | 0.82 | patatin-like protein 2 | 1.14 | 0.013821 | 0.19 | 0.69181 |
| MLOC_30245 | 0.82 | 1.29 | 0.42 | 0.60 | 0.12 | 0.73 | agmatine coumaroyltransferase-2-like | 1.12 | 0.015017 | -0.28 | 0.50549 |
| MLOC_73488 | 0.21 | 0.30 | 0.12 | 0.95 | 0.07 | 0.36 | serine/threonine-protein kinase At5g01020-like | 1.10 | 0.016882 | 0.11 | 0.86783 |
| MLOC_55770 | 0.78 | 0.56 | 0.44 | 0.30 | 0.11 | 0.49 | probable O-methyltransferase 2 | 1.10 | 0.014402 | -0.09 | 0.89474 |
| Novel02265 | 5.38 | 4.70 | 3.08 | 3.69 | 1.56 | 4.82 | GDSL esterase/lipase At5g45910-like | 1.10 | 0.0029212 | 0.02 | 0.97692 |
| MLOC_51311 | 0.14 | 0.05 | 0.03 | 0.13 | 0.00 | 0.22 | G-type lectin S-receptor-like serine/threonine-protein kinase RLK1 | 1.07 | 0.012794 | 0.41 | 0.17247 |
| Novel00367 | 2.78 | 2.18 | 1.64 | 1.47 | 0.94 | 2.73 | zinc transporter 9-like | 1.07 | 0.0018033 | 0.21 | 0.67106 |
| MLOC_60553 | 1.39 | 1.18 | 0.33 | 0.93 | 0.49 | 1.57 | glutamate receptor 2.5-like | 1.06 | 0.012005 | 0.29 | 0.44723 |
| MLOC_25824 | 8.89 | 7.81 | 9.68 | 4.49 | 1.95 | 9.21 | germin-like protein 8-11 | 1.06 | 0.022518 | 0.09 | 0.89185 |
| Novel06235 | 8.93 | 3.10 | 1.57 | 0.95 | 0.91 | 6.58 | glycine-rich RNA-binding protein blt801-like | 1.06 | 0.0221 | 0.33 | 0.40598 |
| MLOC_65056 | 3.39 | 7.19 | 3.04 | 2.89 | 2.38 | 7.39 | E3 ubiquitin-protein ligase EL5-like | 1.05 | 0.011317 | 0.03 | 0.97015 |
| MLOC_34006 | 0.89 | 0.76 | 0.49 | 0.38 | 0.26 | 1.05 | wall-associated receptor kinase 5-like | 1.05 | 0.020471 | 0.21 | 0.68822 |
| Novel02161 | 1.27 | 0.58 | 0.33 | 0.55 | 0.32 | 1.34 | probable serine/threonine-protein kinase At1g18390 | 1.05 | 0.023546 | 0.55 | 0.10282 |
| MLOC_21677 | 1.58 | 3.36 | 1.23 | 2.81 | 0.18 | 0.80 | putative ripening-related protein 5 | 1.04 | 0.024963 | -1.05 | 5.98E-05 |
| MLOC_6793 | 11.52 | 15.16 | 6.22 | 14.98 | 6.71 | 19.56 | uridine 5'-monophosphate synthase-like | 1.04 | 0.010619 | 0.30 | 0.33092 |
| MLOC_58234 | 0.65 | 0.72 | 0.42 | 0.46 | 0.27 | 0.96 | putative wall-associated receptor kinase-like 11 | 1.03 | 0.02019 | 0.19 | 0.71743 |
| MLOC_57610 | 18.99 | 18.64 | 7.98 | 11.37 | 5.40 | 15.51 | scopoletin glucosyltransferase-like | 1.03 | 0.0085359 | -0.17 | 0.75462 |
| MLOC_7557 | 0.99 | 1.24 | 0.69 | 0.87 | 0.45 | 1.38 | E3 ubiquitin-protein ligase ATL6-like | 1.03 | 0.010835 | 0.07 | 0.92253 |
| MLOC_13047 | 3.14 | 2.50 | 1.50 | 1.26 | 0.98 | 3.24 | probable inactive receptor kinase At5g16590 | 1.02 | 0.020482 | 0.20 | 0.70696 |
| MLOC_57245 | 1.04 | 1.76 | 1.07 | 0.56 | 0.11 | 0.70 | protein NRT1/ PTR FAMILY 8.3-like | 1.02 | 0.027783 | -0.33 | 0.34409 |
| MLOC_16287 | 0.67 | 0.44 | 0.42 | 0.32 | 0.08 | 0.51 | UDP-glycosyltransferase 73C6-like | 1.02 | 0.027769 | 0.07 | 0.91165 |
| MLOC_57358 | 7.07 | 9.27 | 7.21 | 10.64 | 4.22 | 11.18 | wall-associated receptor kinase 3-like | 1.02 | 0.0015016 | 0.21 | 0.61045 |
| MLOC_11634 | 1.73 | 3.38 | 1.72 | 2.58 | 0.61 | 2.05 | protein IN2-1 homolog A-like | 1.02 | 0.021229 | -0.35 | 0.39875 |
| MLOC_37693 | 0.24 | 0.51 | 0.28 | 0.51 | 0.22 | 0.73 | F-box protein SKIP27-like | 1.02 | 0.018959 | 0.28 | 0.54714 |
| MLOC_67845 | 0.01 | 0.66 | 0.04 | 0.35 | 0.02 | 0.35 | flavonol synthase/flavanone 3-hydroxylase | 1.00 | 0.024009 | -0.28 | 0.49175 |
| MLOC_59465 | 15.26 | 18.61 | 8.43 | 13.34 | 6.79 | 18.73 | pleiotropic drug resistance protein 3-like | 1.00 | 0.010191 | 0.01 | 0.99037 |
| MLOC_71773 | 2.52 | 3.00 | 1.28 | 2.03 | 1.08 | 3.46 | probable polyol transporter 4 | 1.00 | 0.024574 | 0.12 | 0.83882 |
| MLOC_56446 | 1.26 | 1.51 | 1.51 | 1.84 | 2.05 | 3.83 | cysteine-rich receptor-like protein kinase 10 | 0.66 | 0.0056456 | 1.12 | 3.23E-10 |
| MLOC_67367 | 0.01 | 0.02 | 0.00 | 0.04 | 0.50 | 0.64 | polyubiquitin-like | 0.11 | 0.83865 | 1.39 | 4.59E-09 |
| MLOC_75711 | 0.03 | 0.05 | 0.01 | 0.02 | 0.35 | 0.44 | GDSL esterase/lipase EXL1-like | 0.10 | 0.87485 | 1.00 | 0.00010177 |
| Novel06301 | 6.70 | 11.19 | 5.11 | 7.33 | 30.88 | 36.89 | 7-deoxyloganetin glucosyltransferase-like | 0.05 | 0.91566 | 1.25 | 3.41E-09 |
| Novel00337 | 0.89 | 1.42 | 0.89 | 1.03 | 2.76 | 3.28 | protein BREAST CANCER SUSCEPTIBILITY 2 homolog B-like | 0.04 | 0.85577 | 1.07 | 1.41E-13 |
| MLOC_6543 | 16.16 | 16.53 | 15.07 | 18.32 | 33.91 | 39.85 | clp protease-related protein At4g12060, chloroplastic-like | 0.01 | 0.96277 | 1.19 | 3.12E-26 |
| Novel04096 | 4.90 | 5.19 | 4.83 | 5.64 | 10.30 | 11.86 | clp protease-related protein At4g12060, chloroplastic-like | -0.01 | 0.97969 | 1.10 | 6.60E-18 |
| Novel04533 | 0.61 | 0.15 | 0.19 | 0.13 | 0.81 | 0.92 | probable cadmium/zinc-transporting ATPase HMA1, chloroplastic | -0.01 | 0.98117 | 1.21 | 1.32E-06 |
| MLOC_18281 | 2.71 | 2.49 | 3.17 | 2.76 | 4.80 | 5.42 | probable RNA helicase SDE3 | -0.02 | 0.96494 | 1.03 | 3.32E-15 |
| MLOC_13987 | 1.58 | 1.45 | 1.29 | 1.41 | 4.16 | 4.69 | fanconi anemia group M protein | -0.03 | 0.90701 | 1.55 | 6.33E-36 |
| Novel03400 | 1.76 | 1.68 | 1.39 | 1.26 | 5.72 | 6.25 | probable tRNA modification GTPase MnmE | -0.06 | 0.91014 | 1.27 | 1.51E-08 |
| Novel04538 | 0.45 | 0.24 | 0.23 | 0.19 | 0.96 | 1.04 | probable cadmium/zinc-transporting ATPase HMA1, chloroplastic | -0.07 | 0.88226 | 1.33 | 4.97E-09 |
| Novel03539 | 0.07 | 0.12 | 0.11 | 0.21 | 3.38 | 3.39 | probable tRNA modification GTPase MnmE | -0.13 | 0.84757 | 1.14 | 2.57E-06 |
| MLOC_63821 | 32.66 | 25.50 | 17.70 | 9.98 | 56.21 | 60.48 | chlorophyll a-b binding protein of LHCII type 1 | -0.13 | 0.7498 | 1.06 | 5.07E-10 |
| Novel04537 | 0.41 | 0.11 | 0.26 | 0.14 | 1.04 | 1.02 | probable cadmium/zinc-transporting ATPase HMA1, chloroplastic | -0.15 | 0.78637 | 1.00 | 9.86E-05 |
| Novel02689 | 268.93 | 127.79 | 68.69 | 110.78 | 502.31 | 490.68 | 23 kDa jasmonate-induced protein-like | -0.22 | 0.50437 | 1.39 | 6.16E-11 |
| MLOC_69650 | 0.91 | 1.07 | 0.76 | 0.96 | 3.23 | 3.16 | fanconi anemia group M protein | -0.22 | 0.2898 | 1.34 | 4.26E-17 |
| MLOC_13076 | 0.17 | 0.16 | 0.15 | 0.15 | 7.15 | 6.57 | probably inactive leucine-rich repeat receptor-like protein kinase At3g28040 | -0.33 | 0.2438 | 3.96 | 7.40E-115 |
| Novel02812 | 0.77 | 0.85 | 0.67 | 0.89 | 19.60 | 15.33 | protein FAR1-RELATED SEQUENCE 5-like | -0.38 | 0.44315 | 1.35 | 1.31E-08 |
| MLOC_61767 | 0.44 | 0.74 | 0.34 | 0.79 | 17.50 | 12.98 | protein FAR1-RELATED SEQUENCE 5-like | -0.48 | 0.27098 | 1.64 | 1.84E-12 |
