# Supplementary material for: WRKY Transcription Factors Associated With NPR1-Mediated Acquired Resistance in Barley Are Potential Resources to Improve Wheat Resistance to Puccinia triticina
Source: Front Plant Sci. 2018 Oct 17;9:1486. doi: 10.3389/fpls.2018.01486 (PMC6199750; doi:10.3389/fpls.2018.01486)
Supplement: Supplementary file 10 [file Table_4.docx]

**Supplementary Table S4.** List of Type III DEGs.

| **Gene_id** | **WT_CK** | **WT_PST** | **OE_CK** | **OE_PST** | **Kd_CK** | **Kd_PST** | **Gene Annotation Information** | **log2FoldChange Kd_PSTvsKd_CK** | **padj** | **log2FoldChange Kd_PSTvsWT_PST** | **padj** | **log2FoldChange OE_PSTvsOE_CK** | **padj** | **log2FoldChange OE_PSTvsWT_PST** | **padj** |
| --- | --- | --- | --- | --- | --- | --- | --- | --- | --- | --- | --- | --- | --- | --- | --- |
| Novel05777 | 2.72 | 3.27 | 1.73 | 4.00 | 1.33 | 4.33 | wall-associated receptor kinase 2-like | 1.20 | 0.000884 | 0.31 | 0.37298 | 0.78 | 0.02081 | 0.29 | 0.37833 |
| MLOC_73488 | 0.21 | 0.30 | 0.12 | 0.95 | 0.07 | 0.36 | serine/threonine-protein kinase At5g01020-like | 1.10 | 0.016882 | 0.11 | 0.86783 | 0.46 | 0.26307 | 0.71 | 0.038896 |
| MLOC_63644 | 43.64 | 29.63 | 56.82 | 54.00 | 42.16 | 70.09 | rps8, BrdiC_p061; ribosomal protein S8 | 0.49 | 0.03729 | 1.02 | 4.15E-08 | -0.10 | 0.89916 | 0.77 | 0.00209 |
| MLOC_66134 | 10.45 | 20.45 | 4.19 | 20.52 | 2.52 | 7.28 | probable WRKY transcription factor 70 | 0.91 | 0.040299 | -0.64 | 0.04353 | 1.42 | 1.05E-08 | 0.03 | 0.96697 |
| MLOC_13908 | 12.81 | 41.29 | 4.66 | 20.37 | 4.94 | 17.69 | glucan endo-1,3-beta-glucosidase, acidic isoform-like | 1.00 | 0.028226 | -0.49 | 0.1585 | 1.01 | 0.001248 | -0.49 | 0.20269 |
| MLOC_12709 | 1.47 | 2.94 | 0.70 | 3.37 | 0.45 | 1.43 | cytochrome P450 72A15-like | 1.02 | 0.017591 | -0.58 | 0.078995 | 0.82 | 0.018854 | 0.10 | 0.86108 |
| MLOC_68184 | 29.63 | 59.52 | 31.59 | 88.35 | 12.57 | 60.24 | chitinase 8-like | 1.35 | 0.001236 | 0.01 | 0.98937 | 0.34 | 0.57431 | 0.53 | 0.041293 |
| Novel02253 | 28.73 | 51.82 | 12.03 | 40.17 | 9.65 | 23.85 | ABC transporter C family member 3-like | 0.93 | 0.009683 | -0.74 | 0.008593 | 1.06 | 0.000193 | -0.21 | 0.65956 |
| MLOC_37050 | 30.09 | 65.02 | 11.67 | 37.10 | 12.10 | 26.77 | ABC transporter C family member 3-like | 0.79 | 0.039908 | -0.84 | 0.001609 | 1.02 | 0.000419 | -0.38 | 0.37415 |
