# Supplementary material for: WRKY Transcription Factors Associated With NPR1-Mediated Acquired Resistance in Barley Are Potential Resources to Improve Wheat Resistance to Puccinia triticina
Source: Front Plant Sci. 2018 Oct 17;9:1486. doi: 10.3389/fpls.2018.01486 (PMC6199750; doi:10.3389/fpls.2018.01486)
Supplement: Supplementary file 11 [file Table_5.docx]

**Supplementary Table S5.** A summary of the transient expression assay conducted in the current study.

| **Gene expressed** | **Number of biological replicates** | **Percentage of *Pt* sporulation areas**  **Mean ± SE** | **Combined *P* value** |
| --- | --- | --- | --- |
| AGL1 | 70 | 7.5 ± 0.7 | N. A. |
| *TaPR1b* | 63 | 4.3 ± 0.5 *** | <.0001 |
| *HvWRKY4* | 18 | 10.8 ± 1.5 | 0.5213 |
| *HvWRKY6* | 29 | 3.8 ± 0.9 ** | 0.0002 |
| *HvWRKY17* | 17 | 6.7 ± 1.1 | 0.7423 |
| *HvWRKY19* | 43 | 6.1 ± 0.5 | 0.1653 |
| *HvWRKY20* | 21 | 7.1 ± 1.1 | 0.9726 |
| *HvWRKY31* | 29 | 8.9 ± 1.2 | 0.6454 |
| *HvWRKY40* | 42 | 3.3 ± 0.5 *** | <.0001 |
| *HvWRKY64* | 12 | 5.8 ± 1.3 | 0.6854 |
| *HvWRKY70* | 35 | 1.8 ± 0.2 *** | <.0001 |
| *HvWRKY76* | 14 | 11.9 ± 2.8 ** | 0.0021 |
